# A Tri-part Protein Complementation System Using Antibody-Small Peptide Fusions Enables Homogeneous Immunoassays

**DOI:** 10.1038/s41598-017-07569-y

**Published:** 2017-08-15

**Authors:** Andrew S. Dixon, Sun Jin Kim, Brett K. Baumgartner, Sylvia Krippner, Shawn C. Owen

**Affiliations:** Department of Pharmaceutics and Pharmaceutical Chemistry, College of Pharmacy, University of Utah, 30 South 2000 East, Room 301, Salt Lake City, UT 84112 USA

## Abstract

Protein-fragment complementation is a valuable tool for monitoring protein interactions. In complementation assays, the reporter fragments are directly fused to the interacting proteins, eliminating the possibility of monitoring native interactions. In principle, complementation could be achieved by placing the reporter fragments on antibodies which bind to the proteins of interest, enabling the monitoring of endogenous protein interactions or detection of a single protein in a homogeneous immunoassay. Previous reports have demonstrated proof-of-concept of this approach; however, current complementation systems have not met the practical requirements as suitable fusion partners for antibodies while providing the sensitivity needed for immunoassays. To surmount these challenges, we created a first-in-class, tri-part split luciferase consisting of two 11-residue peptides that are used as the antibody appendages. As an initial proof-of-concept, we used antibody-peptide fusions and found them to be capable of quantifying pg/mL concentrations of soluble or cell-bound HER2, proving this unique complementation system overcomes previous limitations and transforms this approach from merely possible to practical and useful. As shown herein, this dual-peptide system provides a rapid, simple, and sensitive “add-and-read” homogeneous immunoassay platform that can be broadly adapted as an alternative to traditional immunoassays, and in the future should enable complementation to be expanded to monitoring endogenous protein interactions.

## Introduction

Detection and quantification of biomolecules is vital in the health sciences. In a research setting, quantification and characterization of proteins is essential to understanding the mechanistic underpinnings of cellular processes. In a clinical setting, the detection of biomarkers provides a means for disease diagnosis, prognosis, and monitoring therapeutic outcomes. As such, the ability to accurately and reliably detect proteins in a timely manner is highly important.

Immunoassays, such as ELISA, western blot, or immunocytochemistry/immunohistochemistry, are the current standard in protein detection. Since their inception, immunoassays have been advanced by replacing radioisotopes with enzymes^1, 2^, and the addition of multiplexing^3^; however, the general protocol of most immunoassays is tedious. Typical assay operation involves a step to prevent non-specific adsorption of protein (blocking), incubation with a primary antibody, incubation with a labeled secondary antibody, and repeated washes in between each of these steps. Standard immunoassay detection depends entirely on the presence of a signal from a labeled antibody, necessitating the elimination of all unbound antibody. Alternative methodologies that empower single step immunoassays (homogeneous) are greatly beneficial and highly desirable^4^.

The key to eliminating multi-step protocols is a signal that is activated only in the presence of the antigen. Among the methodologies that have been used to achieve an antigen-dependent signal is conjugating donor and acceptor fluorophores to a pair of antibodies and measuring the fluorescence resonance energy transfer (FRET)^5^. When both antibodies are bound to the antigen the fluorophores are held within a distance that allows efficient energy transfer from the donor to the acceptor. Although this approach can support single-step immunoassays, the efficiency of the energy transfer, and consequentially the assay, is highly sensitive to the distance separating the donor and acceptor fluorophores ($efficiency\propto \frac{1}{{r}^{6}}$)^6, 7^. As such, creation of a successful assay is dictated by the ability to achieve the proper distance between the donor and acceptor molecules (<10 nm); a distance that is determined by where the antibodies bind on the antigen relative to each other and where the fluorophores are conjugated on the antibodies. Furthermore, the analysis of the energy transfer can be challenging and requires proper instrumentation, technique, and controls.

Another approach to generate an antigen-specific signal is through luminescent singlet oxygen channeling^8^ (Alpha Technology including AlphaLISA and Alpha Screen). In this approach, one antibody is linked to a photosensitizer that produces singlet oxygen when irradiated with light at 680 nm (donor), and the other antibody is linked to a molecule that reacts with singlet oxygen to produce chemiluminescence (acceptor). As the singlet oxygen produced by the donor particle is a transient species, only the acceptor particles in a sandwich-like complex with antigen and donor particles will be excited and produce luminescence. This method is more versatile than FRET, however some limitations include light sensitivity of the donor particles, temperature sensitivity, and interference of singlet oxygen by hemoglobin. Possibly the greatest limitation of this method, however, is the requirement for an intense light source (laser) at 680 nm which fluorescent plate readers in most laboratories do not have.

In addition to the previously mentioned drawbacks, current methods for homogeneous immunoassays require conjugation of the antibody. The requirement of chemical conjugation increases the labor and cost to produce the assay reagents, and adds a potential source of lot-to-lot variability. Nevertheless, chemical conjugation is also beneficial in some settings, particularly when the unmodified antibody is readily available or when the sequence of the antibody is unknown. Conversely, recombinant antibody production is relatively inexpensive and simple, but requires known antibody sequences. Ideally, the homogeneous immunoassay methodology would be amenable to chemical conjugation *and* creating genetic fusions for maximum flexibility and to enable the benefits of each to be exploited as the situation dictates. Creating genetic fusions to fluorescent proteins for FRET is possible, and has been used for some homogeneous immunoassays, however the sensitivity demonstrated is not sufficient for widespread use with all antigens. An improved method is still needed, and the ability to use either genetic fusions or perform chemical conjugation would be of great interest. To this end, we sought to create an alternative homogeneous immunoassay wherein the necessary reagents could easily be produced in most laboratories, including chemical synthesis and conjugation or use of genetic fusions, and exhibits an output that is easy to measure (including accessible instrumentation), all in a robust platform that is amenable to a variety of antigens.

Protein complementation (also referred to by numerous other names such as protein-fragment complementation assay (PCA)^9, 10^, split protein complementation^11^, or bimolecular fluorescence complementation (BiFC)^12, 13^, is typically used for studying protein-protein interactions and is an interesting alternative for generating an antigen-dependent signal achievable with genetic fusion proteins. In these assays, a reporter (typically an enzyme or fluorescent protein) is inactivated by splitting it into two fragments. Although each fragment is inactive alone, when placed together they refold into the active reporter. Just as protein-protein interactions can be monitored by fusing each fragment to a pair of interacting proteins, a homogeneous immunoassay could be created by fusing the fragments to a pair of antibodies that each bind the target antigen. In such a composition, antigen binding brings the fragments together and produces the active reporter, which quantitatively indicates the presence of the antigen. In contrast to protein interaction assays where the fusions are used inside cells, the fusions used in an immunoassay would need to be purified and stored until the time of use. As such, the complementation system needs to function efficiently outside the highly concentrated cellular environment and be a suitable antibody partner to facilitate the expression, purification, and storage of the assay reagents.

A pioneering report by Ghosh and colleagues demonstrated antigen detection with antibodies fused to a split Firefly luciferase^14^. Importantly, the low functionality of the split system outside of the cellular context was surmounted by co-translating the antibody fusions with protein disulfide isomerase and using lysates from a cell-free expression in place of purified components. This report validates complementation has potential for creating homogeneous immunoassays. The next hurdle to overcome in using complementation in immunoassays is the ability to readily express, purify and store the antibody fusions in order to meet practical assay requirements. Accordingly, there is a need to retool complementation systems to satisfy the unique requirements of immunoassays, and a system comprised of peptides would be keenly suited for enabling facile production of genetic fusions as well as their chemical synthesis and conjugation to the antibodies.

**Figure 1:**
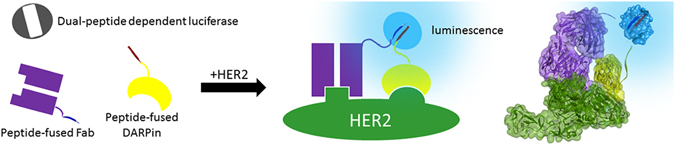
Scheme of Target Engaged Complementation (TEC), utilizing a reporter split into inactive components. Two fragments of the split reporter are each fused to an individual binding proteins such as an antigen binding fragment (Fab) or Designed Ankrin Repeat Protein (DAPRin) that bind specifically to a non-overlapping domain of a target protein. As the two target proteins interact, the antibody-fusions are forced into proximity, inducing enzyme-complementation and luminescent activation.

A promising alternative to using large reporter fragments as fusions is a ternary system such as that created with green fluorescent protein (GFP)^15^. This system consists of one large “detector” fragment that is not fused to either of the proteins being studied and two small peptides which are used as the fusion partners of the interacting proteins. As opposed to other reporter fragments, the peptides are minimal appendages to the proteins of interest and reduce the risk of generating problems during expression, purification, and storage; yet, the formation of the fluorescent complex is slow (hours) and is unsuitable for rapid antigen detection. However, it is worth noting that a potential advantage of GFP may be found in the irreversible complex formation, which could enhance sensitivity. Inspired by the peptide-based ternary GFP, we sought to create a ternary system that would combine the desirable attributes of a peptide-based system with rapid complex formation, high sensitivity (strong signal with minimal background), and a large dynamic range- properties that can be provided by a luciferase. A recent report from Dixon, *et al*. describing NanoLuc Binary Technology (NanoBiT)^16^ is particularly interesting because one of the NanoBiT components is an 11-amino acid peptide and is based on the extremely bright NanoLuc luciferase (100-fold brighter than Firefly^17^). In order to develop a ternary luminescent system, we further dissected NanoLuc into two 11-amino acid peptides and a third, 16.5 kDa “detector” protein. This ternary system is uniquely suited for homogeneous immunoassays we refer to as Target Engaged Complementation (TEC) (Fig. 1) by providing a strong assay output that is easy to measure and uses minimal antibody appendages that can readily be produced recombinantly, purified, and stored for future use. Alternatively, the 11-amino acid peptides can be synthesized and chemically conjugated to the antibodies, providing a highly flexible system for creating immunoassays in a variety of ways depending on the user needs. These desirable attributes enable the general application of this methodology to a variety of antibodies and other binding proteins.

For the development and validation of TEC reported here, we selected epidermal growth factor receptor 2 (ErbB-2, or HER2) as it is an established and highly characterized biomarker^18–21^. HER2 is the focus of significant research efforts, reflecting the therapeutic implications stemming from its overexpression in cancers of the breast^22^, ovaries^23, 24^, uterus^25, 26^, gastric tract^27, 28^, and lung^29^. While HER2 cell-surface expression levels are used to diagnose cancer^30^, detection of the HER2 extracellular domain shed into the blood stream can be used for monitoring response to treatment and recurrence of disease^31–33^. Therefore, the ability to rapidly quantify HER2 levels, both in tissue samples and in serum, is of clinical importance. We demonstrate the ability of TEC to accurately quantify HER2 concentrations using a simple one step method using genetic fusions. We selected HER2, and genetic fusions as opposed to chemical conjugation, to explore the feasibility of the concept; nevertheless, TEC can potentially be applied to the detection of any antigen of interest, and be performed with antibodies conjugated with the peptides. Furthermore, the luminescence generated with this dual peptide complementation system provides a signal that is measurable by instrumentation accessible to almost every laboratory. As such, TEC has the potential to simplify and expand the applications of immunoassays across disciplines.

## Results

### Development of peptide-based complementation system

**Figure 2:**
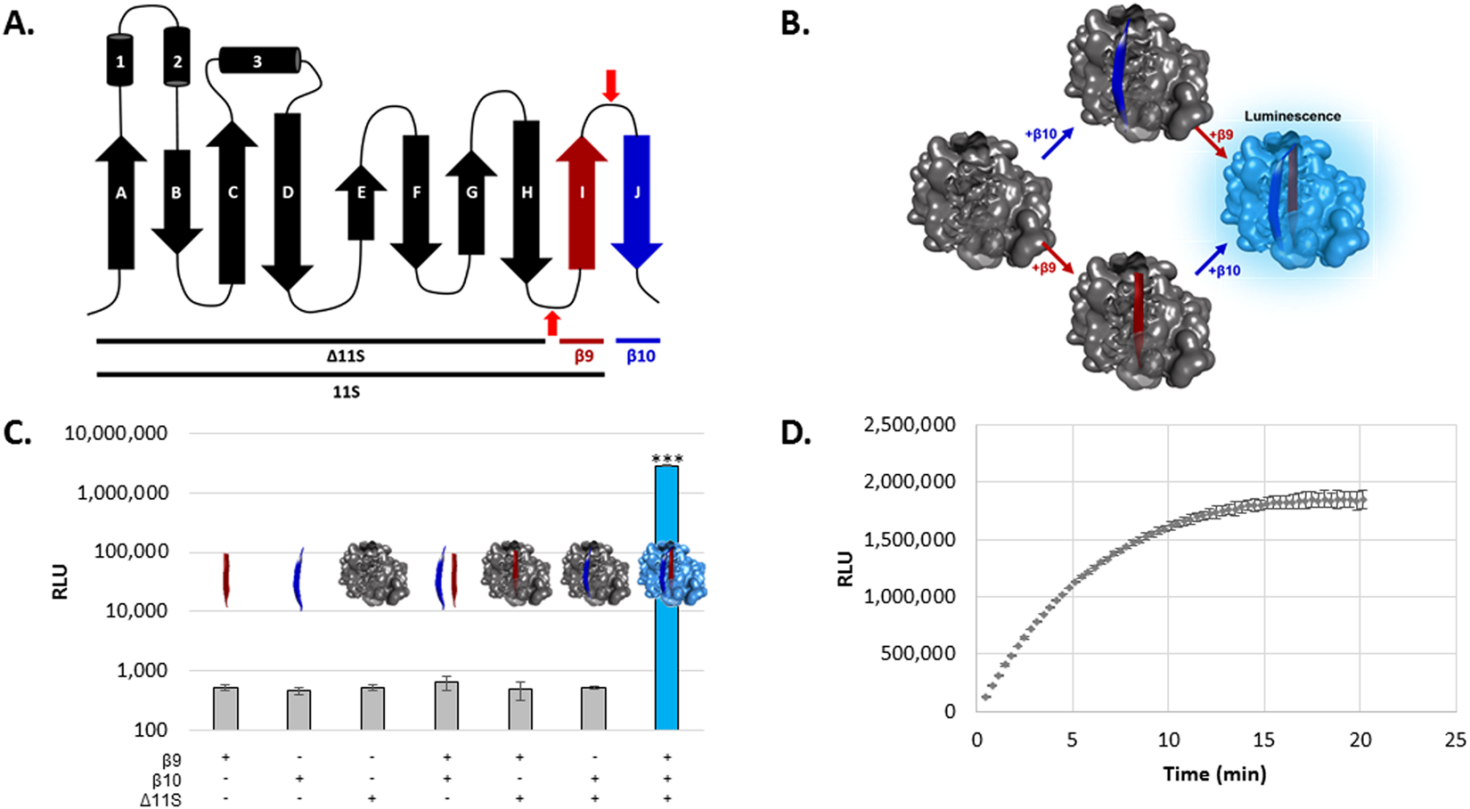
Design of a dual peptide complementation system. (**A**) Topological representation of the 10 β-stranded structure of NanoLuc (adapted from Dixon *et al*.^16^ with permission from ACS Chem. Bio.). The ternary system consists of strands 1–8 (Δ11S) and two 11 amino acid peptides stemming from strands 9 (β9, red) and 10 (β10, blue). Sequences of Δ11S, β9, and β10 are listed in supplemental information, Table S1. (**B**) Structural model illustrating the activation of Δ11S only in the presence of both β9 and β10. Model was created from PDB ID 5IBO. (**C**) Luminescence produced from different combinations of the complementation components. Luminescence is only produced with all three components, ***p < 0.001. Concentrations of β9, β10, and Δ11S were 1 μM, 1 μM and 1:10^5^ dilution of lysate, respectively. (**D**) The luminescent signal over time following the addition of Δ11S (1:10^5^ dilution of lysate) to a solution of 1 μM β9 and β10. Under these conditions, full complementation occurs in under 20 minutes.

Although using complementation to monitor protein interactions inside cells has proven extremely useful, few demonstrations of complementation with purified fragments have been achieved. Successful implementation of TEC requires purified antibody fusions that can be stored and configured as assay reagents. As such, we designed a complementation system aimed at meeting the stringent requirements of a TEC assay, and the use of peptides as the antibody fusion partners is key in this design. The recently described “NanoBiT” complementation system provided the starting point for peptide-based complementation^16^. As reported by Dixon, *et al*., NanoBiT is derived from the parent luciferase, NanoLuc - a 10-stranded beta-barrel protein. The main fragment, termed “11S”, is comprised of beta-strands 1–9 and the other piece is an 11-amino acid peptide which stems from the tenth beta-strand (Fig. 2A – to simplify nomenclature, we use “β10” to refer to this peptide stemming from the tenth β-strand). We further dissected and modified NanoLuc into beta-strands 1–8 (termed Δ11S), and two peptides originating from strands 9 and 10 (termed β9, and β10, respectively. See Fig. 2A,B for illustration and Table S1 for amino acid sequences). This dissection of NanoLuc produced a complementation system such that each of the TEC antibodies needs only an 11-amino acid appendage and should facilitate their production, storage, and use.

We hypothesized that activation of Δ11S occurs only when both β9 and β10 are in close proximity (Fig. 2B). The high local concentration driven by the protein interaction in standard complementation assays, or by antibodies targeting adjacent epitopes as projected in TEC, was mimicked by high concentrations (μM) of synthetic β9 and β10 (Fig. 2C). As predicted, the mixture of all three components is the only combination that produced significant luminescence. Δ11S (used as an unpurified lysate diluted 1:20 throughout this work except where specifically stated) was not active alone nor when mixed with either of the individual peptides. Furthermore, the luminescence emission of the ternary system (Supplementary Figure S1) was essentially identical to that of the binary system and the parental NanoLuc^16, 17^, indicating dissecting NanoLuc in this manner did not significantly alter its luminescent properties. The K_M_ was also compared and found to be only slightly higher than the binary system from which it was derived (3.7 μM for the ternary system vs 2.6 μM for NanoBiT, Figure S2). Of note, lower than expected luminescence was observed in PBST at substrate concentrations higher than ≈20 μM for both the binary and ternary systems. These higher concentrations were not used in calculating the K_M_ values. Surprisingly, unlike previous reports where protein dissection results in a loss of activity, Δ11S was capable of producing the same amount of luminescence as an equimolar amount of 11S (Figure S3). It is important to note, that the loss commonly observed for complementation systems is upon splitting a full protein. Here, we have split an already fragmented protein. With the exception of tripartite GFP, this has never been done before. Unfortunately, the ternary GFP was not compared to the predecessor binary GFP, leaving it uncertain as to whether similar activity should be expected or if this is a unique property of our ternary system. Regardless, these properties (luminescence spectrum, K_M_, and luminescence intensity) indicate the ternary system maintains characteristics to the binary system from which it was derived. As the ability to rapidly form the active luminescent complex is critical for optimal detection, we monitored the luminescent signal following the addition of Δ11S into 1 μM β9 and β10 and found that 90% of the maximal signal was obtained in ≈10 minutes under these conditions, demonstrating the rapid formation of active complex (Fig. 2D).

### Molecular Modeling and Screen to Identify Optimal Antibody Pairs

**Figure 3:**
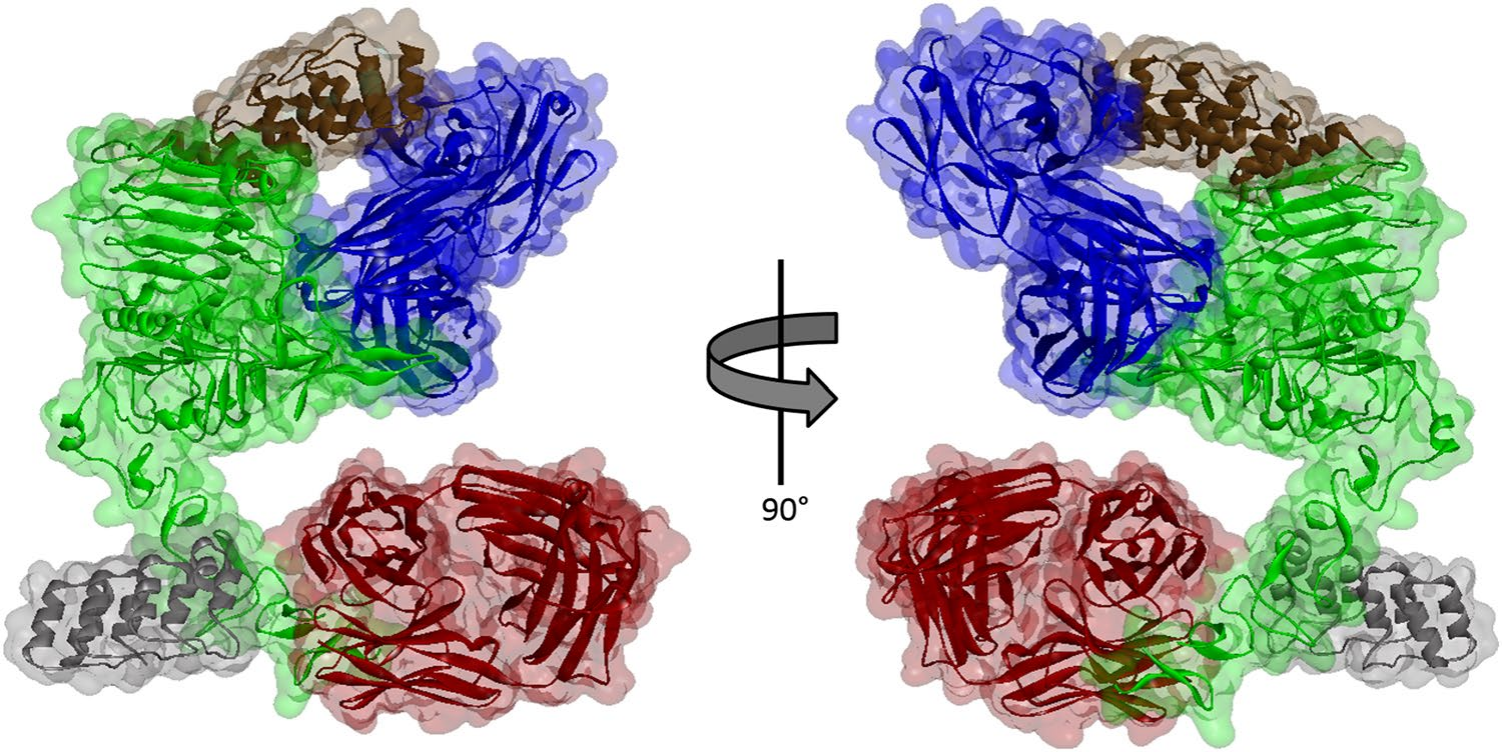
Molecular model of HER2 bound by two antibodies and two DARPins. The extracellular domain of HER2 (green) from four different crystal structures were superimposed to model the spatial proximity between the various binders. Trastuzumab is represented in red (PDB ID 1N8Z), Pertuzumab in blue (PDB ID 1S78), DARPin G3 in gray (PDB ID 4HRN), and DARPin 9.29 in brown (PDB ID 4HRL).

To achieve TEC, a pair of antibodies binding proximal, but non-overlapping epitopes are required. Numerous antibodies and other binders have been crystallized bound to HER2 and are available in the Protein Data Bank (PDB)^34–39^. We selected four HER2 binders based primarily on their binding of distinct HER2 epitopes by superimposing the HER2 portion of respective crystal structures (Fig. 3, see Supplementary Figure S4 for superimposition of the various crystal structures). The set consists of two antibodies (trastuzumab and pertuzumab) and two Designed Ankyrin Repeat Proteins (DARPins, DARPin G3 and DARPin 9.29). Trastuzumab and pertuzumab are both FDA-approved drugs for the treatment of HER2-positive breast cancer^40, 41^. As evident from the superimposition of their structures, each binds distinct HER2 domains: trastuzumab binds HER2 domain IV which is close to the cell surface and does not interfere with HER2 oligomerization^42^, while pertuzumab binds the protruding knob of domain II - an important interface for dimerization^34^. Like trastuzumab, DARPin G3 also binds domain IV^36^, but binds the side distal to the trastuzumab binding site. DARPin 9.29 binds domain I adjacent to pertuzumab^36^. Importantly, all four of these proteins bind HER2 with high affinity (Supplemental Table T2, K_D_ values between 0.09–3nM^36, 43–47^). As such, the elementary modeling of these binders guided us to speculate that any two of them could yield a functional TEC pair. In addition to these binders with characterized epitopes, we also included in the set an antibody, 73 J, which has not been crystallized, and while it does not compete with trastuzumab for binding^43^, the exact epitope is unknown.

To screen for functional TEC pairs, we designed several constructs consisting of each peptide fused to the N- and C-terminus of all five binders. For the antibodies, a Fab format was used, providing the opportunity to make a fusion at either the light or heavy chain termini. By selecting one of each of the N- and C-termini we achieved a reasonable set for screening with sufficient opportunities for identification of functional pairs (10 fusions with β9 × 10 fusions with β10 = 100 possible pairs). We installed an 18 amino acid linker between the binder and the peptide to add spatial flexibility. If fully extended, this linker is capable of spanning ≈65 Å, suggesting the termini of the binding pair need to be within ≈130 Å; the modeled distance between pairs of termini in our set are 43–141 Å (Supplementary Table S2).

Each of the 10 β9-fusions were screened against the 10 β10-fusions (100 total pairs). Each pair was screened on HER2^+^ SKOV3 cells: the cells were incubated with the binder pair simultaneously at ambient temperature, washed, and Δ11S lysate was added along with the substrate and the luminescence was measured. The average signal-to-background from this screen performed on three different days is summarized in Table 1 (see Supplementary Figure S5 for full results). We found that 23 pairs produced a signal at least 100-fold higher than the background, indicating functional TEC assays could be obtained from almost any binding pairs given their epitopes are distinct. Moreover, the ability to use 73 J for TEC demonstrates a successful assay can be created with an antibody without specific knowledge regarding the binding epitope site. Although any of these pairs could potentially be optimized and configured for the efficient detection of HER2, we selected only the top two pairs, β9-L73J:G3-β10 and β9-9.29:PertH-β10, for detailed characterization and analysis.

**Table 1:** Screen of 100 pairs of binders to identify functional TEC pairs.

	β9-9.29	9.29-β9	β9-G3	G3-β9	β9-LPert	PertH-β9	β9-HTras	TrasH-β9	β9-L73J	73 JH-β9
β10-9.29	13 ± 13	1 ± 1	4 ± 4	12 ± 6	3 ± 1	31 ± 28	38 ± 26	5 ± 6	93 ± 72	4 ± 2
9.29-β10	99 ± 49	5 ± 3	122 ± 84	75 ± 48	265 ± 137	346 ± 135	40 ± 4	29 ± 14	597 ± 352	13 ± 5
β10-G3	36 ± 13	11 ± 12	7 ± 3	28 ± 12	32 ± 8	21 ± 20	87 ± 45	15 ± 10	197 ± 80	21 ± 14
G3-β10	19 ± 12	31 ± 27	12 ± 7	93 ± 37	353 ± 63	103 ± 62	508 ± 108	118 ± 58	**1358 ± 45**	47 ± 29
β10-LPert	2 ± 1	2 ± 2	2 ± 0	3 ± 1	0 ± 0	0 ± 0	3 ± 2	1 ± 1	1 ± 1	1 ± 1
PertH-β10	**671 ± 28**	345 ± 130	65 ± 29	122 ± 80	4 ± 3	11 ± 4	211 ± 81	128 ± 31	367 ± 221	38 ± 17
β10-HTras	167 ± 153	2 ± 1	5 ± 1	45 ± 26	108 ± 78	11 ± 12	80 ± 49	6 ± 4	7 ± 2	18 ± 13
TrasH-β10	44 ± 22	60 ± 45	33 ± 26	61 ± 39	114 ± 104	40 ± 29	11 ± 7	6 ± 3	83 ± 71	9 ± 8
β10-L73J	173 ± 208	9 ± 4	21 ± 23	126 ± 102	4 ± 4	12 ± 11	3 ± 1	6 ± 3	2 ± 1	3 ± 1
73JH-β10	81 ± 52	39 ± 26	219 ± 91	95 ± 35	23 ± 9	60 ± 37	170 ± 43	25 ± 12	6 ± 3	7 ± 2

HER2-expressing SKOV3 cells were incubated with clarified lysate of the binders for 1 hr, washed 3 times, and the luminescence measured 30 min after addition of Δ11S lysate (diluted 1:20) and Nano-Glo substrate (10 μM). Values are averages of the signal-to-background ratio from screens performed on three separate days ± the standard deviation. The name of the binder indicates the fusion location and orientation, e.g. β10-LPert is Pertuzumab with β10 fused to the N-terminus of the light chain, whereas PertH-β10 is Pertuzumab with β10 fused at the C-terminus of the heavy chain. Cells with bold text are the top two pairs identified in screen.

### Validation Using Purified Protein

Next, we purified the fusions to enable the optimization and configuration of TEC. The design of the fusions included a His-tag, and the four fusions comprising the top two pairs were purified using standard IMAC procedures. Although long term storage stability is beyond the scope of this proof-of-concept study, the purified proteins retain ≥90% TEC activity from the originating lysate and have been stored (in solution at −80 °C) for several months without a noticeable loss in activity. For this initial manuscript, we focused on assessing the feasability of TEC. As such, using lysates of Δ11S is sufficient. Nevertheless, see below for an initial purification of Δ11S and use thereof. Although each of the parent binders has been extensively validated to bind HER2 in previous studies, we verified the binding of these purified fusions through an indirect ELISA (Figure S6), and confirmed the peptides did not significantly alter the binding properties of the antibody.

**Figure 4:**
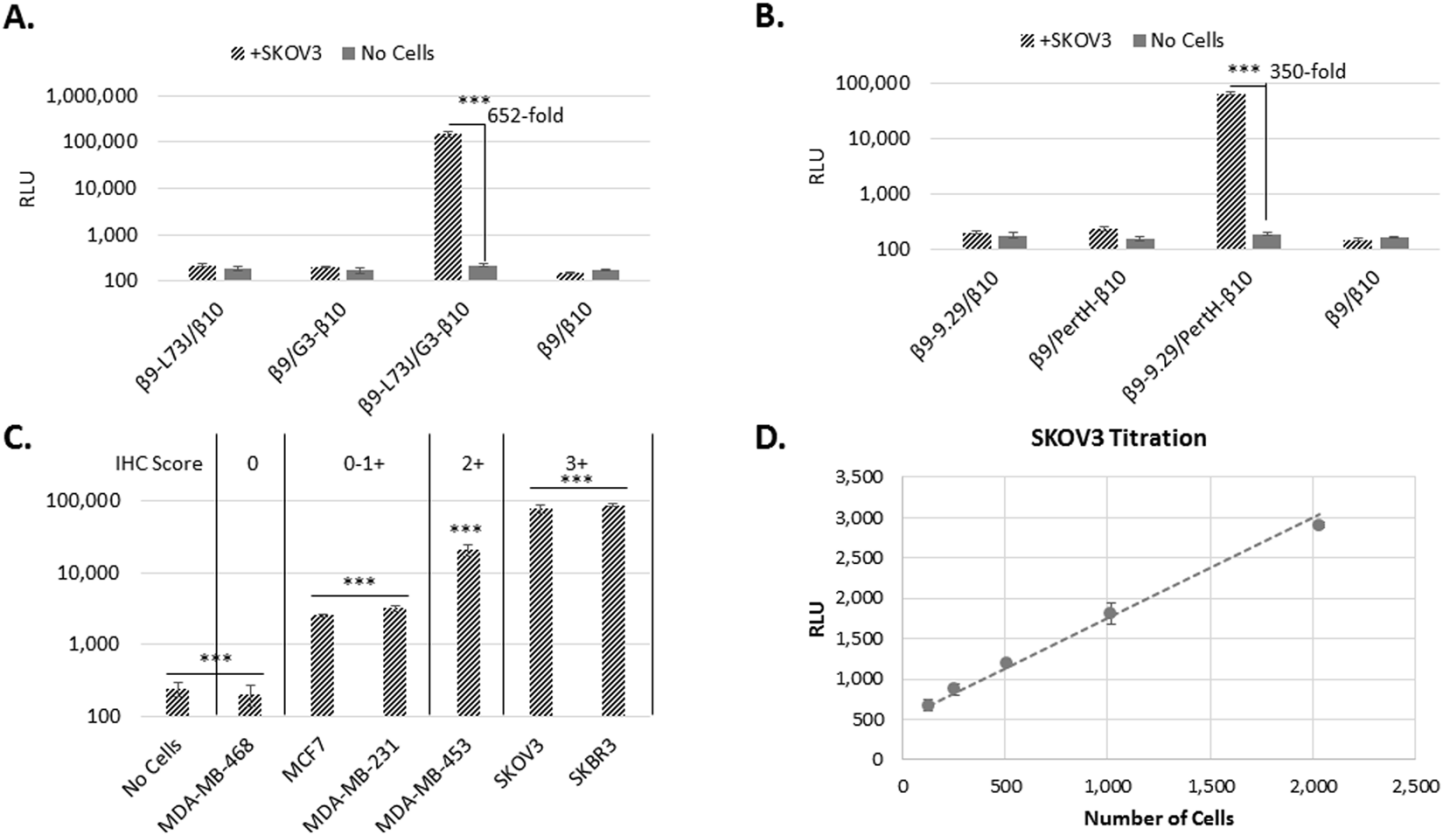
Validation of TEC using purified antibody fusions. TEC with the combination of β9-L73J and G3-β10 (**A**) or β9-9.29 and PertH-β10 (**B)**. None of the fusions alone, nor the combination of synthetic β9 and β10 peptides added at the same concentration as the fusions produced significant luminescence. Assay was performed as in the screen of 100 pairs except 100 nM purified binders were incubated with the cells. (**C**) TEC signal (normalized to signal from Cell-titer Glo 2.0 to account for cell number) obtained by incubating with 100 nM β9-L73J/G3-β10 for 1 hr, washing 3 times, adding Δ11S/Nano-Glo substrate (1:20 diluted lysate and 10 μM, respectively) on various cell lines. Reported immunohistochemistry (IHC) scores for HER2 expression are indicated at top of graph. (**D**) Varying numbers of SKOV3 cells were incubated for 1 hr with a homogeneous TEC detection solution (12.5 nM β9-L73J, 2.5 nM G3-β10, 1:20 dilution of Δ11S lysate, and 10 μM Nano-Glo substrate). The limit of detection was determined to be 125 cells. All data are averages from three replicate wells with the error bars indicating the standard deviation, ***p < 0.001.

We repeated the HER2 detection assay on SKOV3 cells as before (incubate cells with binders, aspirate and detect with Δ11S and substrate) using the purified fusions and included non-targeted β9 and β10 to validate that the signal observed is generated as a result of directing the peptides to HER2 (Fig. 4A,B). When each of the fusions were assayed with the complementary non-targeted peptide, negligible signal was obtained. These signals are comparable to that obtained with the pair of targeting fusions in the absence of HER2 (no cells). Only with both of the targeting fusions, and in the presence of HER2, is the luminescent signal produced. These results confirm the functionality of these TEC pairs and demonstrate the ability to achieve TEC using purified fusions, a significant advancement in the development of a TEC assay.

### Differentiation of HER2 Expression Level on Cells

We measured the TEC signal on a panel of breast cancer cells lines (and one ovarian line) with various levels of HER2 (Fig. 4C, and Supplemental Figure S7 for analysis of cell viability) to further probe the correlation between signal production and HER2 expression. The luminescent signal observed corresponds with reported expression profiles of HER2 in these cells. The TEC signal also matches traditional scoring of HER2 expression by IHC, indicating that TEC could be used as a simple, fast, and quantitative method for profiling HER2 expression. However, this potential is largely dependent on the number of cells required to produce a TEC signal. To assess this question, we serially diluted SKOV3 cells into a homogeneous TEC detection solution (Fig. 4D), and determined that only 125 cells are needed for detection in a TEC assay. That a signal can be obtained from this small number of cells is promising that TEC could be used for quantifying the expression level of relevant biomarkers in patient samples.

### Quantification of Soluble HER2

Following the identification of functional TEC pairs, we aimed to configure TEC into a one-pot detection solution capable of detecting antigen with a single addition of the analyte. As opposed to the previous validations of TEC with HER2 expressed on the surface of adherent cells where unbound antibodies were washed away prior to detection, this solution based approach consists of all components necessary to generate luminescence (β9-binder, β10-binder, Δ11S, and substrate). Configuring this solution requires sufficient antibody to bind antigen while maintaining a low concentration of the peptides to minimize complementation (and background signal) in the absence of the antigen. We found that low nM concentrations of antibody-fusion proteins are optimal for solution based TEC. This solution enabled the detection of HER2+ cells with a single addition (Fig. 4D). The results obtained with this complete TEC solution are very similar to those obtained by separating the binding and detection steps, and validate their use in a one-step immunoassay (add and read).

**Figure 5:**
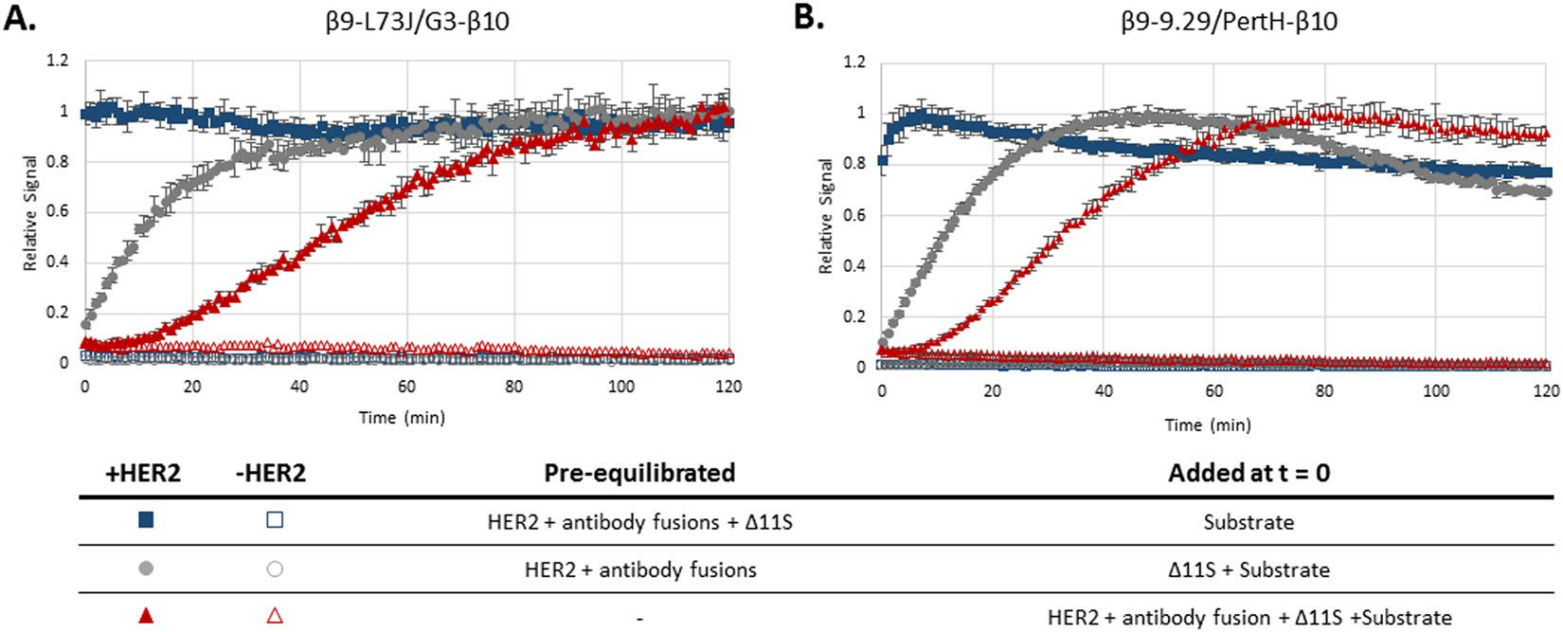
Signal Kinetics of TEC. (**A**) The signal kinetics with the combination of β9-L73J (12.5 nM)/G3-β10 (2.5 nM) or (**B**) β9-9.29 (2 nM)/PertH-β10 (20 nM). The kinetics of the single-step TEC assays are compared to two different conditions pre-equilibrated with HER2: antibody fusions (gray circles), or antibody fusions and Δ11S (blue squares). The difference between the pre-equilibrated solutions indicate the kinetics of binding between antibodies and HER2 and between the peptides and Δ11S. Δ11S lysate was diluted 1:20 and Nano-Glo substrate was 10 μM in all experiments.

Antigen binding is necessary in all immunoassays. For most immunoassays, each antibody is incubated with antigen for at least 1–2 hours to allow the binding reaction to reach equilibrium. In TEC, where all assay components are added at once, the binding kinetics will dictate the opportune time for measuring the luminescence. In addition to antibody binding, Δ11S also needs to bind β9/β10 and form the active luciferase. We performed kinetic studies to ascertain the timing for signal measurement, and to differentiate the antibody binding from the formation of the luciferase. As shown in Fig. 5, when all components (β9- and β10-fused binders and Δ11S) are pre-equilibrated with HER2, luminescence is obtained immediately. If Δ11S is not pre-equilibrated, but rather added with substrate to pre-equilibrated antibodies, the luminescent signal increases with time in accordance with the formation of the luciferase complex. Interestingly, the formation of the luciferase is slower for the 73 J/G3 combination, reaching 90% of the maximal signal at ≈50 minutes as compared to ≈30 minutes for 9.29/pertuzumab. Among other possibilities, this difference in rate may be a reflection of a larger distance separating the fused termini when bound to HER2. Unfortunately, the epitope bound by 73 J has not been fully characterized, preventing the confirmation of this hypothesis through structural modeling. When nothing is pre-equilibrated with HER2, a further time delay is observed in the rise of the signal, consistent with the requirement of the antibodies to bind HER2 and Δ11S to bind β9/β10 and form the active luciferase. In the absence of any pre-equilibration, as would be applicable to a homogeneous immunoassay, these kinetic profiles demonstrate that the maximal signal for both TEC pairs is obtained within two hours. Furthermore, for 9.29/pertuzumab, a signal-to-background (S/B) of five is obtained within 20 minutes, providing proof-of-principle that a one-pot detection solution can be both simple and fast. Naturally, the assay kinetics are dependent on the concentration of the antigen - assay kinetics for various concentrations of HER2 within the detection range for both β9-L73J/G3-β10 and β9-9.29/PertH-β10 are included in the next section.

### Detection Range of HER2

Although simplicity and speed are valuable assay attributes, they are worthless without being coupled to sensitivity. We used recombinant HER2 (extracellular domain) to determine the sensitivity limitations of the homogenous TEC assay. In accordance with the Clinical and Laboratory Standards Institute (CLSI) guidance EP17, we use the following definitions outlined by Armbruster and Pry^48^:

1 $$Limit\,of\,Blank\,(LoB):\,meanRL{U}_{blank}+1.645\times S.D{.}_{blank}$$

2 $$Limit\,of\,Detection\,(LoD):LoB+1.645\times S.D{.}_{lowconcentrationsample}$$

**Figure 6:**
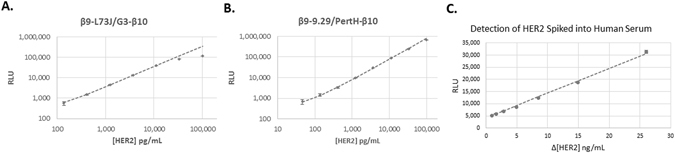
The linear range and limit of detection for homogeneous HER2 TEC assays. For these assays, a complete detection solution was used consisting of the β9- and β10-fused binders, Δ11S lysate diluted 1:20, and 10 μM Nano-Glo substrate. (**A**) For β9-L73J (12.5 nM)/G3-β10 (2.5 nM), the limit of detection was determined to be 137 pg/mL, and the signal was linear up to 11 ng/mL. (**B**) For β9-9.29 (2 nM)/PertH-β10 (20 nM), the limit of detection was determined to be 45 pg/mL, and the signal was linear up to approximately 100 ng/mL. (**C**) Demonstration that TEC can be used to detect HER2 in 100% human serum. The TEC signal was linear down to a change of sub-ng/mL concentrations (Δ = 900 pg/mL), p < 0.001 between response for 9.8, 10.7, 11.4, and 12.5 ng/mL HER2. In general, human serum contains a normal HER2 level of ~9.8 ng/mL, preventing the analysis of a “blank” sample. 15 ng/mL is the established threshold separating normal for elevated HER2 levels.

We defined the LoD as the lowest concentration explicitly measured, and did not extrapolate to a concentration not specifically tested. For 73 J/G3, the LoD was 137 pg/mL and the linear range extended up to a concentration of 11 ng/mL (Fig. 6A); however, the combination of 9.29 and pertuzumab was found to exceed these capabilities in terms of both the limit of detection and the linear range (Fig. 6B). The LoD for this pair was determined to be 45 pg/mL and the signal was linear up to 100 ng/mL. The signal kinetics for each concentration of HER2 is provided in Supplemental Figure S8. That the 9.29/pertuzumab combination outperformed 73 J/G3 was surprising based on results obtained on HER2 expressing cells, and may suggest 73 J/G3 can drive the complementation when each is bound to separate HER2 molecules associated in a homodimer (present on the SKOV3 cells, but not when recombinant HER2 extracellular domain is used) as well as a single molecule of HER2. Together, these results indicate β9-L73J/G3-β10 is our best pair for measuring HER2 expression on cells, while β9-9.29/PertH-β10 is the best pair for measuring shed HER2. A previous demonstration using complementation to detect HER2 reported the ability to detect 500 pM HER2^14^. Our results demonstrate the ability to detect sub-picomolar concentrations (45 pg/ml = 0.7 pM), an improvement of approximately 700-fold. More importantly, commercially available ELISA kits report sensitivities in the range of 8–24 pg/mL with linear ranges up to approximately 5 ng/mL, indicating TEC is comparable in sensitivity and linear range to standard ELISA methods and is achieved in a fraction of the time and labor. For a comparison to a standard ELISA for HER2, see Supplemental Figure S11. Moreover, the sensitivity obtained here is comparable to the sensitivity reported for the commercially available AlphaLISA HER2 kit (Perkin Elmer, 54.1 pg/mL), and can be performed on a standard plate reader instead of a specialized instrument needed for the AlphaLISA (AlphaLISA technology requires a laser capable of excitation at 680 nm and is not typically found on basic fluorescent plate readers).

We also sought to compare the ternary system to the binary system from which it was derived, NanoBiT. To do so, the NanoBiT components were genetically fused to the best pair of binders for detection of soluble HER2, DARPin 9.29 and pertuzumab. The fusions were created in a way such that the fusion points and orientations would be the same as already identified for this pair of binders; β10 at the C-terminus of the pertuzumab heavy chain, and 11S at the N-terminus of DARPin 9.29 (the β9 portion of 11S is at the C-terminus and is connected to 9.29 just as in β9-9.29). Detection of HER2 was also possible with the binary system, as seen in Supplemental Figure S8. While the binary system achieved a similar level of sensitivity as the ternary (LoD = 411 pg/mL), the linear range was much smaller, with the upper limit around 4 ng/mL. This is in stark contrast to the ternary system, which was found to be linear up to around 100 ng/mL, and may represent an advantage of having the largest luciferase component (Δ11S) not directly connected to the binders, allowing the concentration to be independently held high. The assay kinetics were also evaluated (Supplemental Figure S9), and the binary system was found to respond slightly faster to the addition of HER2 as might be expected based on the formation of the enzyme from two pieces instead of three.

### Measuring Serum Concentrations of Soluble HER2

The concentration of soluble HER2 in the bloodstream is currently used for monitoring both treatment efficacy and the recurrence of cancer^31, 33, 49–51^. Therefore, we sought to determine if TEC could be used to accurately detect HER2 in human serum. Through a standard ELISA, the concentration of HER2 in commercially available human serum (Lonza) was found to be 9.8 ng/mL (Supplemental Figure S11), a value consistent with reported levels of HER2 in serum from healthy persons^31^. The threshold for elevated HER2 in serum has been established at ≥15 ng/mL^51^. Based on this threshold and normal serum levels, the assay would need to differentiate small increases in HER2 concentration. To ascertain if TEC has this capability, we spiked HER2 into the human serum with 9.8 ng/mL HER2 already present and performed a TEC assay using the combination of 9.29 and pertuzumab. As seen in Fig. 6C, this TEC assay was capable of differentiating as little as 0.9 ng/mL HER2, and was linear in the applicable concentration range. This demonstrates the ability of TEC to differentiate between biologically relevant concentrations of HER2 in human serum in a rapid and simple manner.

### Purification of Δ11S

The third component of the system, the detector protein Δ11S, is a truncation of 11S. While Δ11S is not appended to either of the binders, it still needs sufficient stability to enable it to be expressed, purified, stored, and used. For this initial work contained in this manuscript, with the primary goal of assessing the possibility and potential of TEC, we viewed Δ11S as a component that may need some protein engineering *if* results indicated it was warranted. As such, a lysate of Δ11S is used throughout this work. As reported herein, the results are indeed promising and justify exploration of the feasibility of purifying Δ11S. To this end, Δ11S was purified using standard IMAC procedures. As seen in Supplemental Figure S12, Δ11S can be purified with a simple His-tag, without the need for any additional solubility/expression tag, and similar in procedure to the purification of 11S but with a lower yield. To assess its activity, the purified Δ11S was tested in a TEC assay at various concentrations and compared to the lysate (Supplemental Figure S13). Using the purified protein, it was possible to obtain similar results to those obtained with the lysate, indicating Δ11S can be used as purified protein. While the ability to purify and use Δ11S will be important, it is noteworthy that no significant problems were encountered using the lysates. That lysates stored either at 4 °C or −20 °C could be used for many months without noticeable loss in activity indicates a reasonable level of stability atypical of proteins that have been fragmented, and indicates that for situations where purified proteins are not required, a lysate of Δ11S could be a reasonable option.

## Discussion

We constructed a complementation system that requires two 11-amino acid peptides for the production of luminescence, and use these peptides as antibody fusions in a homogeneous immunoassay that directly links antigen binding to signal production. Performing immunoassays with this peptide-based complementation system is an interesting and beneficial approach. While fragmented proteins typically used for complementation assays can be challenging to use outside of a cellular environment, including expression, purification, and storage, the peptide-antibody fusions used here were readily purified and used successfully as complementation reagents. The use of these small unstructured peptides in place of larger protein fragments plays a significant role in our success because it reduces potential problems commonly associated with large, unstructured protein fragments. The ability to express, purify, and store the antibodies for future use is essential for immunoassays, and the results reported here indicate this complementation system enabled the creation of fusions that meet this practical requirement.

We chose to validate TEC against the known biomarker HER2; however, we envision the same platform can be used against other targets. The use of genetic fusions of small peptides with antibodies enabled us to produce the assay reagents recombinantly in E. coli - a low-cost, simple manner. Nevertheless, the use of genetic fusions, as demonstrated herein, is limited to antibodies with known sequences. In addition to the ability to perform TEC with genetic fusions, the small peptides of this complementation system can also be chemically synthesized and site-specifically conjugated to antibodies (unpublished data), including antibodies of various formats and sources. As such, this approach to homogeneous immunoassays is versatile and should be adaptable to a range of binding proteins and target antigens.

Here we demonstrate how two anti-HER2 binders can be configured to detect HER2. While the detection and quantification of biomarkers is clinically important, TEC has potential beyond single molecule detection. In the same fashion, TEC could be achieved using a pair of antibodies that bind two different molecules, enabling the detection or monitoring of native protein interactions, including homo- and hetero-oligomerization. Intriguingly, when HER2 expressed on cells was used in the screen, the best TEC pair identified (73 J/G3) differed from the best pair when using recombinant HER2 that is monomeric (9.29/pertuzumab). This suggests oligomerization of HER2 is likely contributing to the resulting complementation with 73 J/G3. Additionally, pairs consisting of fusions to the same binder (e.g. β9-9.29/9.29-β10) should not be capable of detecting a monomer as there is only one epitope on each HER2 molecule. As such, results from our screen indicate β9-9.29/9.29-β10 and G3-β9/G3-β10 are pairs that are likely detecting dimeric HER2. Although further work is needed to identify and optimize TEC pairs for measuring HER2 oligomerization, these results suggest TEC can be configured for the detection of protein interactions, including oligomerization. Monitoring interactions of native proteins in biological samples greatly expands the utility of the TEC assays. The sensitivity of TEC reported here is sufficient for detecting HER2, including in 100% human serum, and is comparable to commercially available ELISA kits. This sensitivity is achieved through the formation of a single luciferase for each pair of antibodies and might be improved if signal amplification can be achieved. Signal amplification is typically achieved in immunoassays by increasing the number of reporter molecules per antigen - by using polyclonal antibodies or by conjugating multiple reporter proteins onto the antibody. Similarly, incorporation of multiple peptides onto each antibody may be a means to augment signal and increase the sensitivity of TEC. Among the possible ways to accomplish this is to create genetic fusions with multiple repeats of the peptide, fuse the peptide to more than one terminus, or chemically conjugate multiple peptides to the antibody. Future work will explore these possibilities of increasing the complementation per antibody pair, and may prove valuable for other antigens or circumstances where increased sensitivity is required.

## Conclusion

Immunoassays are essential components of every biomedical laboratory. As such, performing these assays can consume significant time and resources. We engineered an immunoassay where quantitative data is obtained with minimal time, effort, and cost – both in terms of reagent production and performing the assay. Such a unique complementation system, based on two small peptide-fusions, enables facile production of reagents required for a novel homogeneous immunoassay, wherein the need for immobilization, blocking, and washing are eliminated. While the configuration shown here is most similar to an ELISA, this methodology may have promise in immunocytochemistry and immunohistochemistry as well. Importantly, TEC retains the sensitivity required to detect biologically relevant concentrations of antigen. The simplicity, speed, and sensitivity obtained with readily available laboratory instrumentation suggests that TEC using this novel ternary luciferase system is a fertile immunoassay platform.

## Methods

### Cell lines, reagents, and instrumentation

All cell lines were obtained from the American Type Culture Collection (ATCC). Growth media, each supplemented with 10% FBS and 1% Penicillin/Streptomycin, for the cells were as follows: SKOV3 - DMEM, MDA-MB-468 – DMEM +1% MEM non-essential amino acids, MDA-MB-231 – RPMI 1640 + 0.1% Gentamicin, MDA-MB-453 - DMEM, SKBR3 – McCoy’s 5a Modified, and MCF7 – RPMI 1640 + 0.1% Gentamicin +4 mg/mL Insulin. Cells were maintained in 37 °C/5% CO_2_. Recombinant HER2 ECD with C-terminal His tag was purchased from Speed Biosystems and reconstituted in PBS at 100 µg/mL. SHuffle T7 Express chemically competent E. coli cells were purchased from New England Biolabs. Cobalt Agarose Beads (High Density) and HTC Cartridges were purchased from Gold Biotechnology. Nano-Glo, Nano-Glo Live Cell Substrate (5 mM), and Cell-titer Glo 2.0 were purchased from Promega Corporation. Synthetic β9 and β10 peptides were purchased from Peptide 2.0. Protein purification was performed on an AKTA FPLC (GE Healthcare). Luminescence was measured on an Infinite M1000 Pro (Tecan).

### Cloning

cDNA encoding 11S^17^, DARPin 9.29^36^, DARPin G3^36^, VH and VL of trastuzumab^35^, VH and VL of pertuzumab^34^, and 73JFab^43^ were purchased as GeneArt Strings from ThermoFisher Scientific. The VH and VL of trastuzumab and pertuzumab were then appended onto CL and CH1 using the primers indicated in the primer table included in the supplemental material (Supplemental Table S4). The linker, His tag, and β9/β10 peptide were added to the sequences through PCR using primers listed in Supplemental Table S4. Each cDNA was inserted into pF1K T7 (Promega) at the PvuI and XbaI restriction enzyme sites. Δ11S was created by PCR amplifying all except the β9 sequence of 11S and inserting the resulting amplicon into pF1K T7 with PvuI/XbaI. β10Δ11S was created by addition of the β10 sequence onto the forward primer used to amplify Δ11S and inserting the amplicon into pF1K T7 with PvuI/XbaI. Standard genetic engineering protocols were used for all cloning, and each clone was sequence verified.

### Validation of Ternary Complementation System

Solutions of 4 µM β9, 4 µM β10, 1:1 × 10^5^ dilution of Δ11S lysate, and 40 μM Nano-Glo substrate were made in PBS + 0.1% Tween20. In a white 96-well assay plate, 25 µL of each solution or PBST were mixed and incubated at ambient temperature for 30 minutes. Luminescence was then measured on a Tecan Infinite M1000 plate reader using 0.1 Sec integration. Three replicates of each combination were assayed, and values reported are means with standard deviations indicated by the error bars. For kinetic assessment of complex formation, 50 µL of 2 µM β9 and β10 and 20 μM Nano-Glo substrate was mixed with 50 µL of 1:1 × 10^5^ dilution of Δ11S lysate in PBST (0.1%). Luminescence was measured every minute for 20 minutes using an integration of 0.1 Sec.

### Luminescence Emission Spectra

For the binary system, 100 μL of 1 μM β10, 10 μM Nano-Glo substrate, and 11S lysate diluted 1:100 in PBS + 0.1% Tween 20 was added to the well of a white 96-well plate in triplicate. For the ternary system, 100 μL of 1 μM β9 and β10, 10 μM Nano-Glo substrate, and Δ11S lysate diluted 1:4 in PBS + 0.1% Tween 20 was added to the well of a white 96-well plate in triplicate. Luminescent emission was scanned on Tecan Infinite M1000 Pro from 300 nm to 700 nm in 2 nm steps using a bandwidth of 5 nm, integration time of 0.1 Sec, and gain of 122 and 110 for the binary and ternary system respectively.

### Molecular Modeling

A molecular model of trastuzumab, pertuzumab, DARPin 9.29, and DARPin G3 all bound to a single HER2 molecule was created using Discovery Studio 4.0 (Accelrys) and crystal structures obtained from the Research Collaboratory for Structural Bioinformatics Protein Data Bank (RCSB PDB) with the following PDB IDs: 1N8Z (trastuzumab), 1S78 (pertuzumab), 4HRN (DARPin G3), and 4HRL (DARPin 9.29). A HER2 molecule from the structures of pertuzumab, DARPin G3, and DARPin 9.29 was superimposed onto the HER2 from the trastuzumab structure by creating tethers between the α-carbons of the cysteine residues. Using the α-carbon of the terminal residue, the distance between specific termini were then calculated within the created model.

### Screen of 100 TEC pairs

A single colony of SHuffle T7 Express E. coli transformed with plasmid encoding the antibody fusion was used to inoculate a 50 mL LB culture that was grown overnight at 32 °C and 250 rpm. 15 mL of the overnight culture was then diluted into 1 L LB and grown at 32 °C and 250 rpm to an O.D._600_ = 0.6–0.8. IPTG was then added to a final concentration of 0.5 µM and the temperature lowered to 16 °C for overnight expression. Cells were then harvested by centrifuging the culture at 4,700 rpm for 30 minutes at 4 °C. Cell pellets were then suspended in PBS pH 8 (50 mM phosphate, 150 mM NaCl), and sonicated on ice for 2 minutes with 10 second pulses separated by 30 second intervals at 50% amplitude. The lysate was then cleared by centrifuging at 20,000 × g for 1 hour at 4 °C. The concentration of the fusion protein was then assayed through luminescence by mixing 25 µL 11S (1:10^3^) + Nano-Glo Substrate (20 μM) or β10Δ11S (1:105) + Nano-Glo Substrate (20 μM) to 25 µL of various dilutions of the β10-fusion lysate or β9-fusion lysate, respectively. 11S produces luminescence proportional to the concentration of β10, and β10Δ11S produces luminescence proportional to the concentration of β9. As such, each can be used to normalize the concentration of the β10- or β9-fusions in the lysates. A dilution factor for each lysate was determined such that each diluted lysate produces similar luminescence. This dilution factor was used as a crude normalization of the fusion protein used in the screen.

The day after seeding 15,000 SKOV3 cells in the wells of a white 96-well plate, each antibody fusion was diluted (pre-determined dilution factor) into PBS + 1% BSA, and 50 µL of β9-antibody mixed with 50 µL β10-antibody and incubated on cells for 1 hour at ambient temperature with gentle orbital shaking (300 rpm). Antibody fusions were then aspirated and the wells washed three times in 100 µL of PBS + 0.1% Tween20. Luminescence was then measured 30 minutes after the addition of 100 µL Δ11S (1:20) + Nano-Glo Substrate (40 μM).

### Expression and Purification

Expression of the antibody fusions was as described under “Screen of 100 TEC pairs”, using 5mM L-Histidine pH 6 (L-Histidine buffer) in place of the PBS buffer for suspending the cell pellet. A 1 mL HTC cartridge was packed with Cobalt Agarose Beads, equilibrated with L-Histidine buffer, and used for purifying the antibody fusion on an AKTA FPLC. After the clarified lysate was applied to the cartridge and washed with 20 column volumes of buffer, bound protein was eluted with 200 mM imidazole. Fractions collected during the elution phase were analyzed by SDS-PAGE, pooled, EDTA (0.5 mM) Tween20 (0.01%) and Trehalose (0.1%) added, and stored at −80 °C. A Bradford assay (Coomassie Plus Reagent) was performed to determine protein concentration.

#### Δ11S and 11S purification

The plasmids encoding Δ11S and 11S were transformed into SHuffle T7 Express E. coli. 50 mL of LB culture was inoculated with a single colony of E. coli and incubated overnight at 30 °C and 250 rpm. 20 mL of the overnight culture was diluted into 1 L of LB and incubated at 30 °C and 250 rpm until O.D._600_ of 0.5–0.6. Then the culture was induced with 0.4 µM IPTG and the temperature was lowered to 16 °C for overnight culture. Cells were harvested by centrifuging the culture at 4,700 rpm for an hour at 4 °C. After obtaining the cell pellets, they were suspended in PBS pH 8 (50 mM phosphate, 150 mM NaCl) and sonicated on ice for 2 minutes with 10 second pulses separated by 30 second intervals at 50% amplitude. The lysate was then centrifuged at 20,000 × g for 1 hour at 4 °C. The soluble fraction of the lysate was purified using a 1 mL HisPur^TM^ Cobalt Chromatography Cartridge on an AKTA FPLC. For Δ11S, after loading the clarified lysate, the column was washed with 10 mM imidazole for 10 column volumes before gradually increasing the imidazole concentration to 60 mM over 50 column volumes. The imidazole was then increased to 200 mM for 15 column volumes. For the purification of 11S, 20 mM imidazole for 20 column volumes was used to wash the column following loading of the clarified lysate, and then the concentration was ramped up to 60 mM over 20 column volumes. 20 column volumes of 200 mM imidazole were then used to elute any remaining protein. The fractions collected during the purification were analyzed by SDS-PAGE. Fractions with purity estimated to be > 95% were combined and dialyzed against a buffer containing 5 mM L-Histidine, 150 mM NaCl and 0.5 mM EDTA. Following dialysis 0.01% Tween 20 and 0.1% Trehalose were added for storage at −80 °C. The concentration was determined by absorbance at 280 nm and with a Bradford assay (Coomassie Plus Reagent).

### TEC Assays

#### TEC on cells

The day after seeding 15,000 cells in the wells of a white 96-well plate, the media was replaced with 100 μL of 100 nM β9-L73J and G3-β10 and the plate incubated for 1 hour at ambient temperature with gentle orbital shaking (300 rpm). Antibody fusions were then aspirated and the wells washed three times in 100 µL of PBS + 0.1% Tween20. Luminescence was then measured 30 minutes after the addition of 100 µL Δ11S (1:20) + Nano-Glo Substrate (40 μM). The number of viable cells was also quantified using Cell-titer Glo 2.0. At the time of the addition of Δ11S/Nano-Glo substrate, cell growth media was aspirated and replaced with 50 μL plain DMEM. 50 μL of Cell-titer Glo 2.0 was then added to the wells and shaken on an orbital shaker for 2 min. Luminescence was then measured on a Tecan Infinite Pro M1000 with 0.1 Sec integration. The average signal from three replicate wells was then used to normalize the TEC signal obtained from three replicate wells seeded at the same time in the same 96-well plate.

#### Solution-based TEC

The TEC solutions were made in PBS + 0.1% Tween20 and consisted of Δ11S lysate (diluted 1:20), Nano-Glo Substrate (10 μM), and the pair of antibody fusions. The concentrations of the fusions were as follows: β9-L73J – 12.5 nM, G3-β10 – 2.5 nM, β9-9.29 – 2 nM, and PertH-β10 – 20 nM. For assays with recombinant HER2 ECD, 10X TEC solutions were made and 10 µL added to 90 µL of HER2 diluted to concentrations indicated in PBS. For serum assays, 1.05X TEC solutions were prepared and 95 µL added to 5 µL of HER2 diluted to concentrations indicated in 100% human serum. For each well, 2 hrs after addition of the detection solution the luminescence was measured 5 times and averaged.

### ELISA to Quantitate HER2 in Human Serum

A sandwich ELISA was performed using the HER2 ELISA pair set of antibodies from Sino Biological. 100 μL of capture antibody diluted 1:1,000 in PBS pH 7.4 was added to the wells of a clear 96-well ELISA plate and incubated overnight at 4 °C. Wells were then washed with 200 μL TBST (0.05% Tween20) and then blocked with 200 μL of 2% BSA in TBST for 1 hr. Wells were washed with 200 μL TBST before incubation with 100 μL sample or HER2 standard for 2 hrs. Wells were again washed with 200 μL TBST and then 100 μL of detection antibody, diluted 1:200 in TBST + 0.5% BSA, added and incubated for 1 hr. 100 μL of Turbo-TMB ELISA Substrate was then added to each well and incubated for 15 min. before stopping the reaction with 1.8 M H_2_SO_4_. The absorbance at 450 nm was then read on a Tecan Infinite M1000 Pro. All samples and standards were assayed in triplicate.

### Analysis of K_M_

12.5 pM 11S or Δ11S was incubated with 1.25 μM β10 (11S) or 12.5 μM β9/β10 (Δ11S) for 30 min. 40 μL of these solutions were then mixed with 10 μL of Nano-Glo substrate at 5x the concentration indicated (final concentrations in 50 μL volume are 10 pM 11S or Δ11S, 1 μM β10, 10 μM β9/β10, and 1x indicated concentration of Nano-Glo substrate). Luminescence was then measured with 0.1 Sec integration. Data (excluding 27 and 40 μM concentrations) were fit to Michaelis-Menten equation in GraphPad Prism. Highest two concentrations (27 and 40 μM) resulted in lower luminescence, possibly due to solubility, and were not included in fit to determine K_M_ for either 11S or Δ11S.

### Comparison of Luminescence Using Titrations of β9 and β10

For the binary system, 40 μL of 12.5 pM 11S (10 pM final) was mixed with 10 μL of β10 (5x indicated concentration for final 1x indicated concentration) and 50 μM Nano-Glo substrate (10 μM final). Data was fit to the following equation:

3 $$RLU=\frac{RL{U}_{{\rm{\max }}}\times {[\beta 10]}^{h}}{{{K}_{D}}^{h}+{[\beta 10]}^{h}}$$

For the ternary system, 40 μL of 12.5 pM Δ11S (10 pM final) was mixed with 10 μL of β9/β10 (5x indicated concentration for final 1x indicated concentration) and 50 μM Nano-Glo substrate (10 μM final). β9 and β10 were diluted together such that their concentrations are equivalent in all wells. Data was fit to the following equation:

4 $$RLU=\frac{RL{U}_{\max lo}\times {[\beta 9]}^{{h}_{lo}}{[\beta 10]}^{{h}_{lo}}}{{{K}_{D,lo}}^{{h}_{lo}}+{[\beta 9]}^{{h}_{lo}}{[\beta 10]}^{{h}_{lo}}}+\frac{RL{U}_{\max hi}\times {[\beta 9]}^{{h}_{hi}}{[\beta 10]}^{{h}_{hi}}}{{{K}_{D,hi}}^{{h}_{hi}}+{[\beta 9]}^{{h}_{hi}}{[\beta 10]}^{{h}_{hi}}}$$

### Graphing and Statistical Analysis

All statistical analyses were performed using Graph Pad Prism version 5.00 for Windows (Graph Pad Software, San Diego California USA, www.graphpad.com). Differences among groups were assessed by one-way ANOVA with Bonferroni post hoc correction to identify statistical differences among three or more treatments. Alpha levels were set at 0.05 and a p-value of ≤0.05 was set as the criteria for statistical significance. All data are presented as mean ± standard deviation.

## Electronic supplementary material

Supplementary Information 
