# Supplementary material for: A Tri-part Protein Complementation System Using Antibody-Small Peptide Fusions Enables Homogeneous Immunoassays
Source: Sci Rep. 2017 Aug 15;7:8186. doi: 10.1038/s41598-017-07569-y (PMC5557857; doi:10.1038/s41598-017-07569-y)
Supplement: Supplementary file 1 — Supplementary Information [file 41598_2017_7569_MOESM1_ESM.pdf]

Supplementary Information

A Tri-part Protein Complementation System Using Antibody-Small Peptide Fusions Enables Homogeneous Immunoassays

*Andrew S. Dixon, Brett K. Baumgartner, Sun Jin Kim, Sylvia Krippner, and Shawn C. Owen*Author(s), and Corresponding Author(s)**

	Amino Acid Sequence
β9	GSMLFRVTINS
β10	VSGWRLFKKIS
Δ11S	MKHHHHHHMVFTLEDFVGDWEQTAAYNLDQVLEQGGVSSLLQNLAVSVTPIQ<br>RIVRSGENALKIDIHVIIPYEGLSADQMAQIEEVFKVVYPVDDHHFKVILPYGTLVI<br>DGVTPNMLNYFGRPYEGIAVFDGKKITVTGTLWNGNKIIDERLITPD

Table T1. Amino acid sequences of the ternary system.

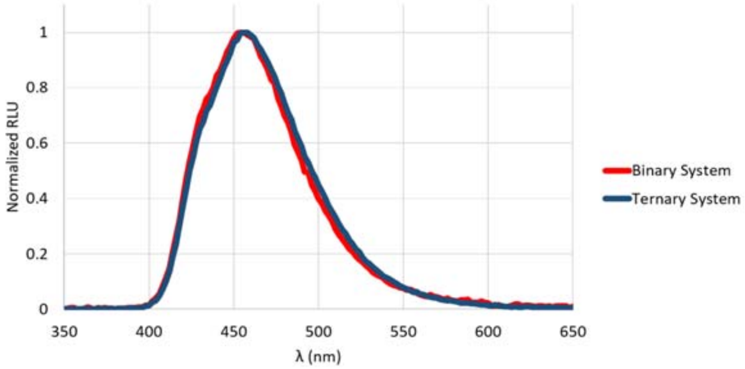

Figure S1. Luminescent emission spectra of the binary and ternary systems. λ_{max} was determined to be 454nm and 458nm for the binary and ternary systems, respectively. 100 μL of 1 μM β10 and 11S lysate diluted 1:100 was used for the binary system, and 1 μM β9, 1 μM β10, and Δ11S lysate diluted 1:4 was used for the ternary system both with 10 μM Nano-Glo substrate. Data represent the averages of three replicates.

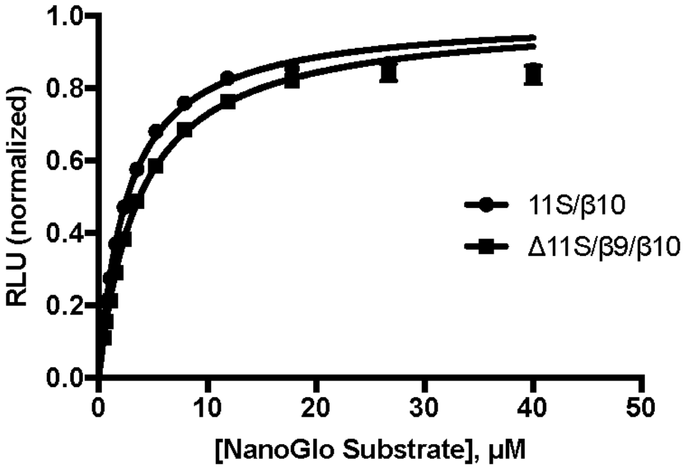

	K_M (μM)	95% CI (μM)
11S/β10	2.6	2.5 to 2.7
Δ11S/β9/β10	3.7	3.6 to 3.9

Figure S2. Analysis of K_M for the binary and ternary systems. Final concentration of 11S and Δ11S was 10 pM incubated with 1 μM β10 or 10 μM β9/β10, respectively, for 30 min prior to addition of Nano-Glo substrate and luminescence measurement.

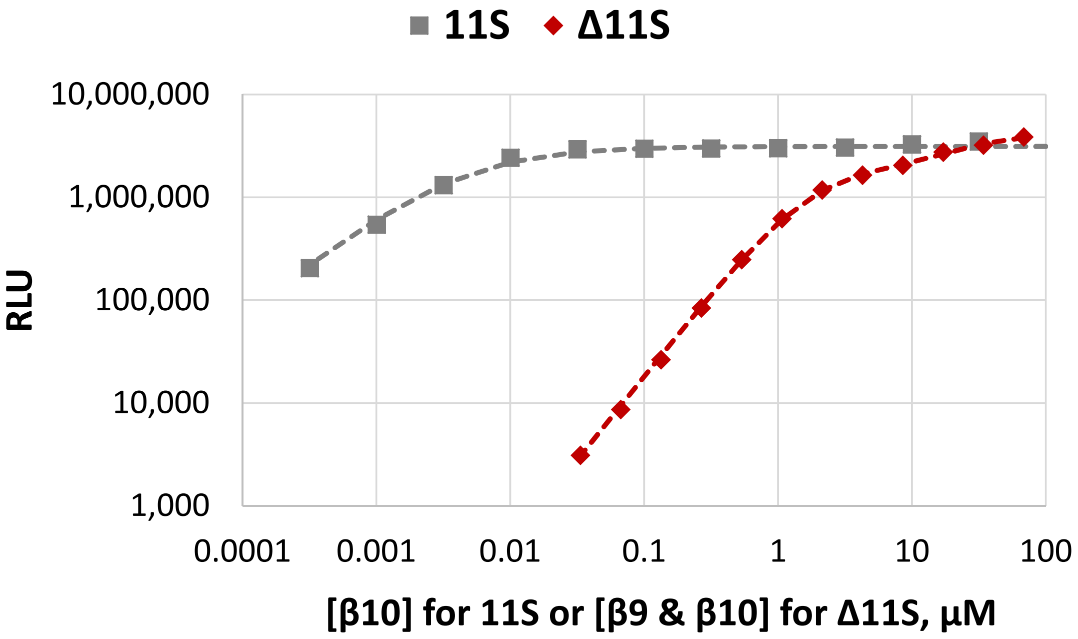

Figure S3. Peptide titrations into 11S and Δ11S. Peptides, β10 for 11S or both β9 and β10 for Δ11S, were titrated into 10 pM concentration of 11S or Δ11S with 10 μM Nano-Glo substrate. Luminescence at high peptide concentrations indicates no loss in luminescence upon splitting off β9 from 11S. Data from 11S titration was fit to 1-site binding equation to give the following values: $RLU_{max} = 3.12 \times 10^6$, $K_D = 4$ nM, h = 1. Data from Δ11S titration was fit to 2-site binding equation to give the following values: $RLU_{max, Lo} = 2.02 \times 10^6$, $K_{Lo} = 2.6\ \mu M^2$, h = 0.81, $RLU_{max, Hi} = 2.02 \times 10^6$, $K_{Hi} = 566\ \mu M^2$, h = 0.99.

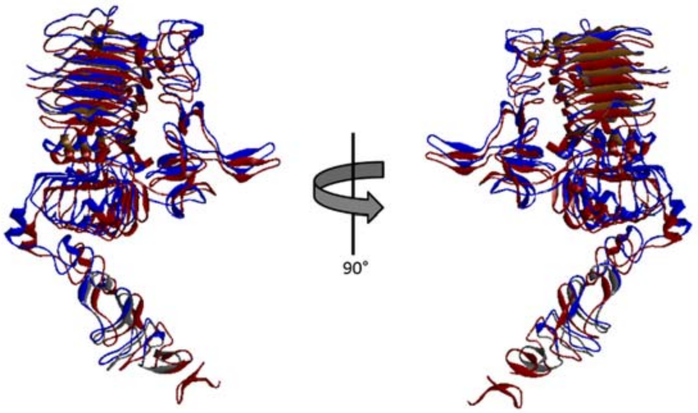

Figure S4. Superimposition of HER2 from four different crystal structures. The HER2 from each structure is colored the same as the binder is colored in Figure 2: PDB ID 1N8Z (Trastuzumab) is red, PDB ID 1S78 (Pertuzumab) is blue, PDB ID 4HRN (DARPin G3) is gray, and PDB ID 4HRL(DARPin 9.29) is brown. For 4HRL and 4HRN, only the domain where binding occurs (domain I and IV, respectively) were crystallized. For 1S78 and 1N8Z, HER2 domains I-IV were crystallized.

Table T2. Reported binding affinities for the binders screened in this work.

**HER2 binder**	**K_D (nM)**	**Reference**
**G3**	0.09	[1-3]
**9.29**	1	[1]
**Tras Fab**	0.5	[4]
**Tras IgG**	0.5	[4]
**73J Fab**	3.26	[4]
**73J IgG**	1.37	[4]
**Pert IgG**	< 0.25	[5]

Table T3. Calculated distances between termini of potential TEC pairs. Molecular modeling was performed using crystal structures deposited in the Protein Data Bank of Trastuzumab (Tras, PDB ID: 1N8Z), Pertuzumab (Pert, PDB ID: 1S78), DARPin G3 (G3, PDB ID: 4HRN), and DARPin 9.29 (9.29, PDB ID: 4HRL) bound to HER2. The HER2 portion from each of the crystal structures was superimposed to model where each one binds HER2 relative to the others. As genetic fusions were to be made, the distances between the termini were measured and are reported in Ångströms, Å.

	β9-LPert	PertH-β9	β9-HTras	TrasH-β9	β9-L73J	73JH-β9	β9-9.29	9.29-β9	β9-G3	G3-β9
β10-LPert	-	-	47	43	-	-	67	56	97	66
PertH-β10	-	-	92	93	-	-	69	52	141	121
β10-HTras	47	92	-	-	-	-	93	100	47	55
TrasH-β10	43	93	-	-	-	-	108	97	111	73
β10-L73J	-	-	-	-	-	-	-	-	-	-
73JH-β10	-	-	-	-	-	-	-	-	-	-
β10-9.29	67	69	93	108	-	-	-	-	102	87

9.29-β10	56	52	100	97	-	-	-	-	132	104
β10-G3	97	141	47	111	-	-	102	132	-	-
G3-β10	66	121	55	73	-	-	87	104	-	-

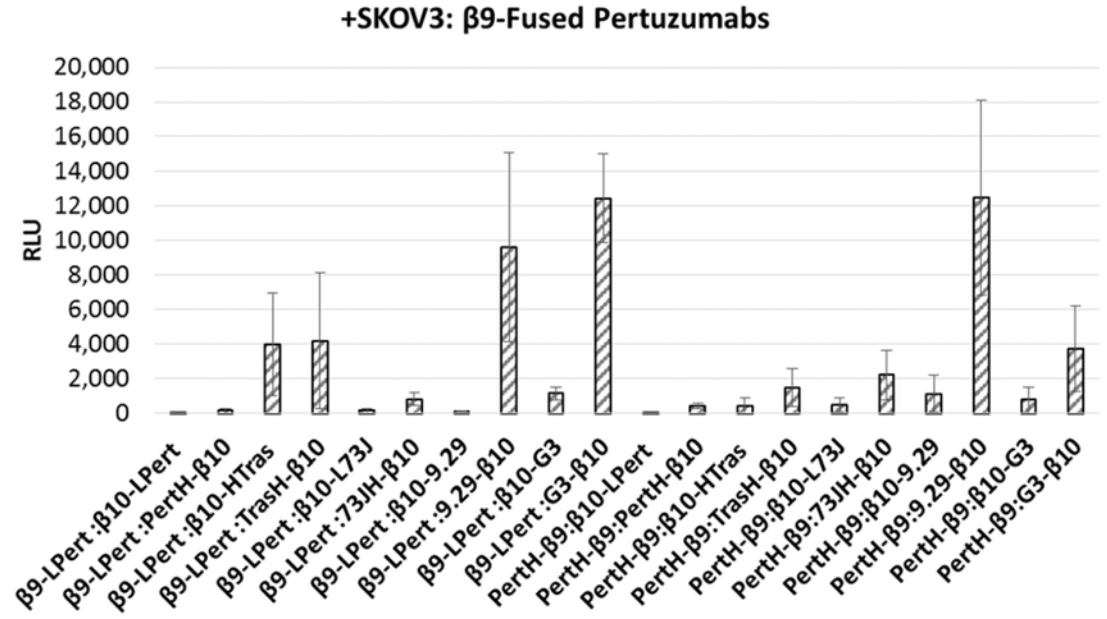
+SKOV3: β9-Fused Pertuzumabs
RLU
20,000
18,000
16,000
14,000
12,000
10,000
8,000
6,000
4,000
2,000
0
β9-LPert:β10-LPert
β9-LPert:PertH-β10
β9-LPert:β10-HTras
β9-LPert:TrasH-β10
β9-LPert:β10-L73J
β9-LPert:73JH-β10
β9-LPert:β10-9.29
β9-LPert:9.29-β10
β9-LPert:β10-G3
β9-LPert:G3-β10
PertH-β9:β10-LPert
PertH-β9:PertH-β10
PertH-β9:β10-HTras
PertH-β9:TrasH-β10
PertH-β9:β10-L73J
PertH-β9:73JH-β10
PertH-β9:β10-9.29
PertH-β9:9.29-β10
PertH-β9:β10-G3
PertH-β9:G3-β10

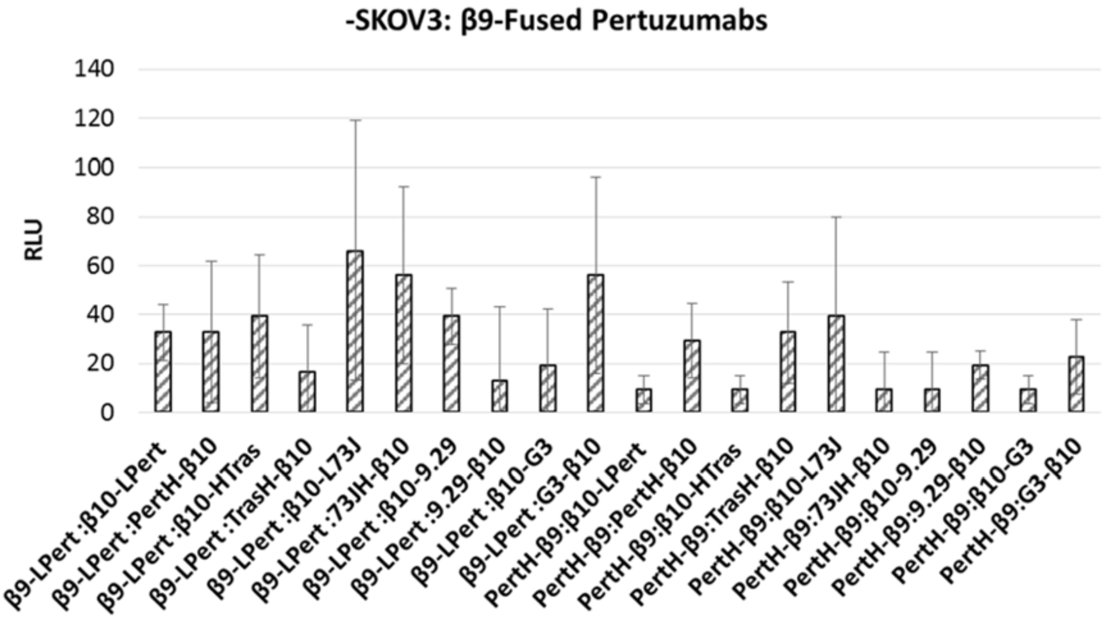
-SKOV3: β9-Fused Pertuzumabs
RLU
140
120
100
80
60
40
20
0
β9-LPert:β10-LPert
β9-LPert:PertH-β10
β9-LPert:β10-HTras
β9-LPert:TrasH-β10
β9-LPert:β10-L73J
β9-LPert:73JH-β10
β9-LPert:β10-9.29
β9-LPert:9.29-β10
β9-LPert:β10-G3
β9-LPert:G3-β10
PertH-β9:β10-LPert
PertH-β9:PertH-β10
PertH-β9:β10-HTras
PertH-β9:TrasH-β10
PertH-β9:β10-L73J
PertH-β9:73JH-β10
PertH-β9:β10-9.29
PertH-β9:9.29-β10
PertH-β9:β10-G3
PertH-β9:G3-β10

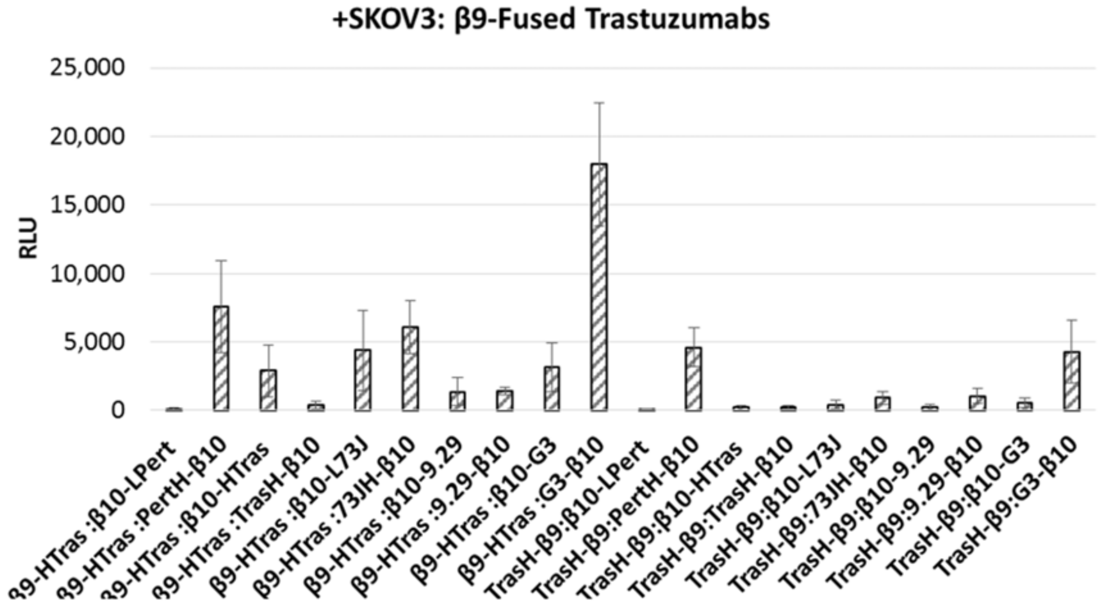
+SKOV3: β9-Fused Trastuzumabs
RLU
25,000
20,000
15,000
10,000
5,000
0
β9-HTras :β10-LPert
β9-HTras :PertH-β10
β9-HTras :β10-HTras
β9-HTras :TrasH-β10
β9-HTras :β10-L73J
β9-HTras :73JH-β10
β9-HTras :β10-9.29
β9-HTras :9.29-β10
β9-HTras :β10-G3
β9-HTras :G3-β10
TrasH-β9:β10-LPert
TrasH-β9:PertH-β10
TrasH-β9:β10-HTras
TrasH-β9:TrasH-β10
TrasH-β9:β10-L73J
TrasH-β9:73JH-β10
TrasH-β9:β10-9.29
TrasH-β9:9.29-β10
TrasH-β9:β10-G3
TrasH-β9:G3-β10

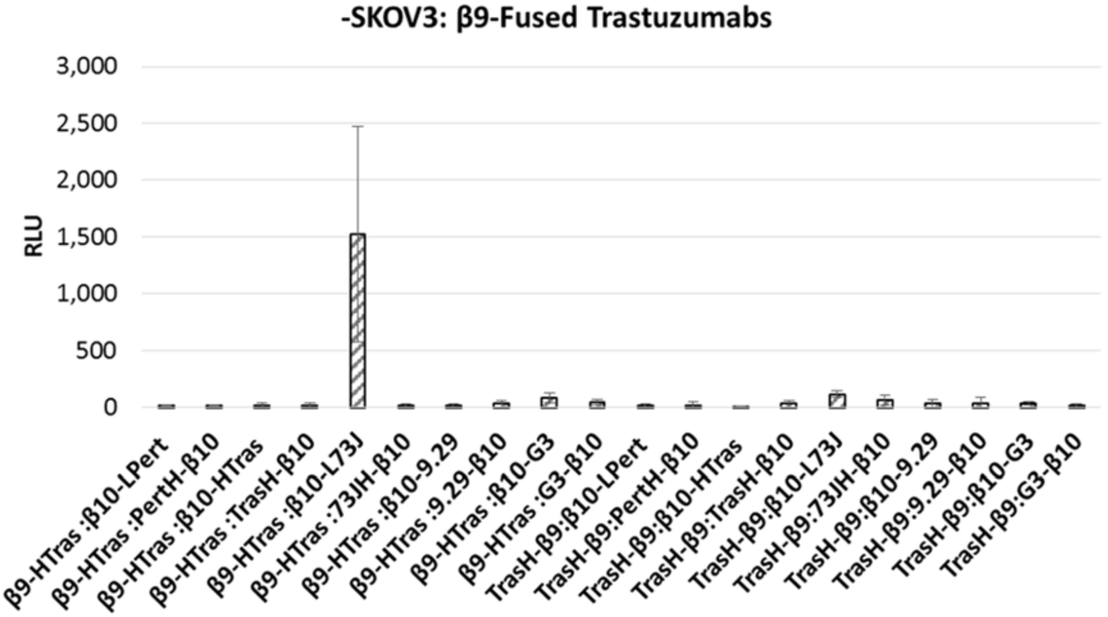
-SKOV3: β9-Fused Trastuzumabs
RLU
3,000
2,500
2,000
1,500
1,000
500
0
β9-HTras :β10-LPert
β9-HTras :PertH-β10
β9-HTras :β10-HTras
β9-HTras :TrasH-β10
β9-HTras :β10-L73J
β9-HTras :73JH-β10
β9-HTras :β10-9.29
β9-HTras :9.29-β10
β9-HTras :β10-G3
β9-HTras :G3-β10
TrasH-β9:β10-LPert
TrasH-β9:PertH-β10
TrasH-β9:β10-HTras
TrasH-β9:TrasH-β10
TrasH-β9:β10-L73J
TrasH-β9:73JH-β10
TrasH-β9:β10-9.29
TrasH-β9:9.29-β10
TrasH-β9:β10-G3
TrasH-β9:G3-β10

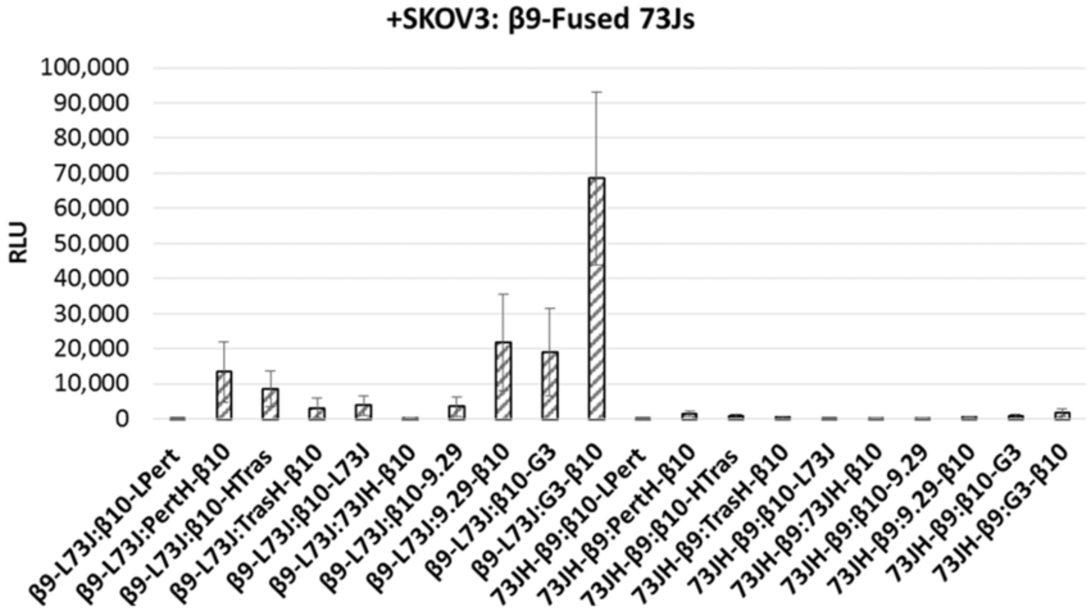
+SKOV3: β9-Fused 73Js
100,000
90,000
80,000
70,000
60,000
50,000
40,000
30,000
20,000
10,000
0
RLU
β9-L73J:β10-LPert
β9-L73J:PertH-β10
β9-L73J:β10-HTras
β9-L73J:TrasH-β10
β9-L73J:β10-L73J
β9-L73J:73JH-β10
β9-L73J:β10-9.29
β9-L73J:9.29-β10
β9-L73J:β10-G3
β9-L73J:G3-β10
73JH-β9:β10-LPert
73JH-β9:PertH-β10
73JH-β9:β10-HTras
73JH-β9:TrasH-β10
73JH-β9:β10-L73J
73JH-β9:73JH-β10
73JH-β9:β10-9.29
73JH-β9:9.29-β10
73JH-β9:β10-G3
73JH-β9:G3-β10

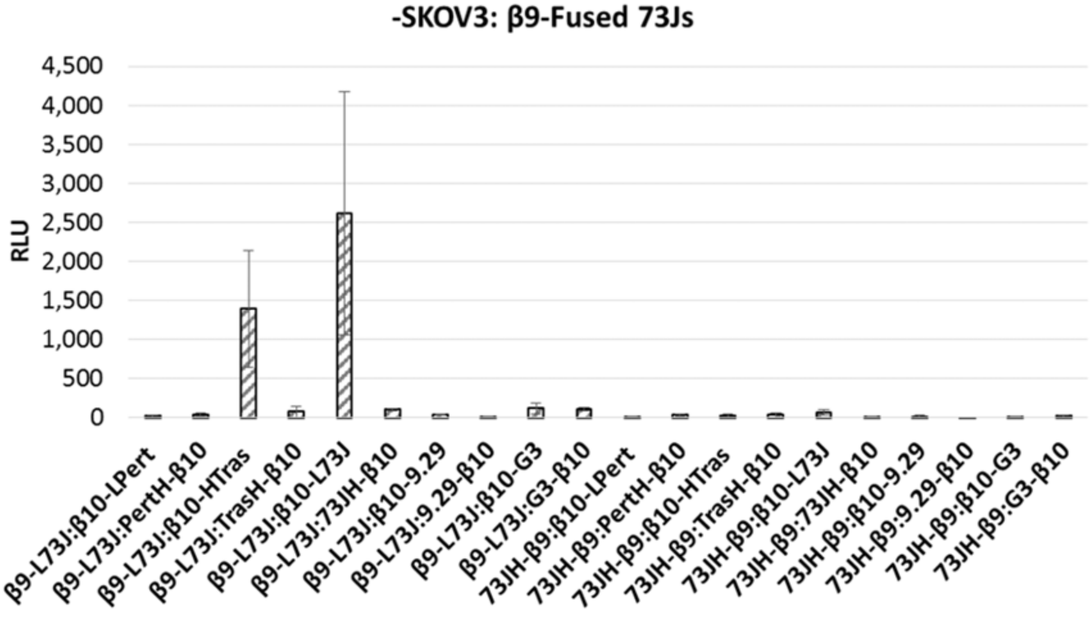
-SKOV3: β9-Fused 73Js
4,500
4,000
3,500
3,000
2,500
2,000
1,500
1,000
500
0
RLU
β9-L73J:β10-LPert
β9-L73J:PertH-β10
β9-L73J:β10-HTras
β9-L73J:TrasH-β10
β9-L73J:β10-L73J
β9-L73J:73JH-β10
β9-L73J:β10-9.29
β9-L73J:9.29-β10
β9-L73J:β10-G3
β9-L73J:G3-β10
73JH-β9:β10-LPert
73JH-β9:PertH-β10
73JH-β9:β10-HTras
73JH-β9:TrasH-β10
73JH-β9:β10-L73J
73JH-β9:73JH-β10
73JH-β9:β10-9.29
73JH-β9:9.29-β10
73JH-β9:β10-G3
73JH-β9:G3-β10

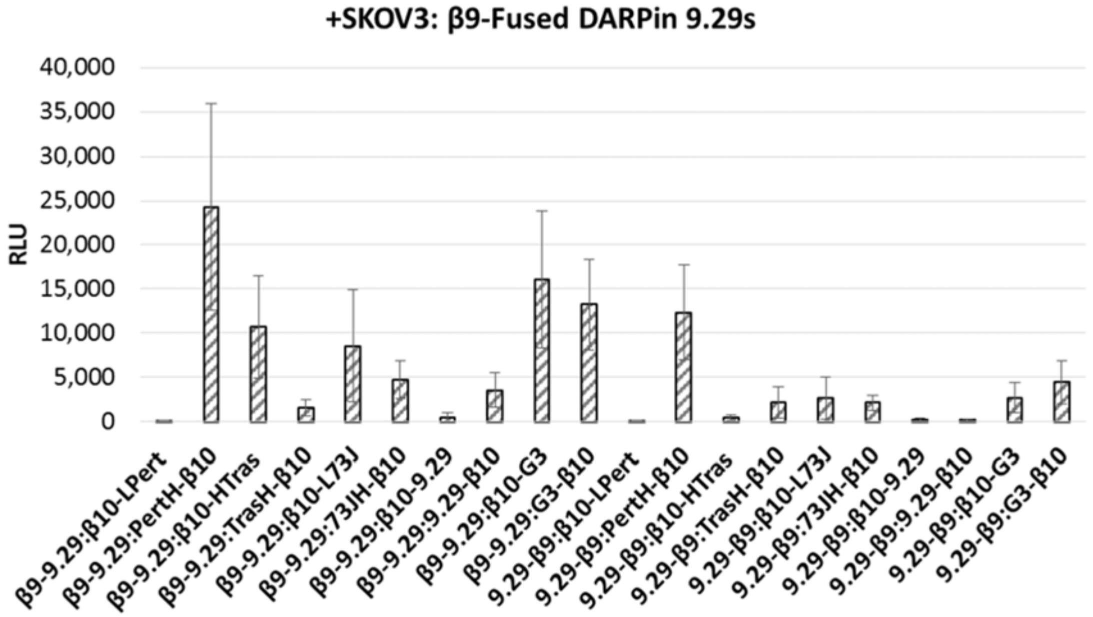
+SKOV3: β9-Fused DARPin 9.29s
RLU
40,000
35,000
30,000
25,000
20,000
15,000
10,000
5,000
0
β9-9.29:β10-LPert
β9-9.29:PertH-β10
β9-9.29:β10-HTras
β9-9.29:TrasH-β10
β9-9.29:β10-L73J
β9-9.29:73JH-β10
β9-9.29:β10-9.29
β9-9.29:9.29-β10
β9-9.29:β10-G3
β9-9.29:G3-β10
9.29-β9:β10-LPert
9.29-β9:PertH-β10
9.29-β9:β10-HTras
9.29-β9:TrasH-β10
9.29-β9:β10-L73J
9.29-β9:73JH-β10
9.29-β9:β10-9.29
9.29-β9:9.29-β10
9.29-β9:β10-G3
9.29-β9:G3-β10

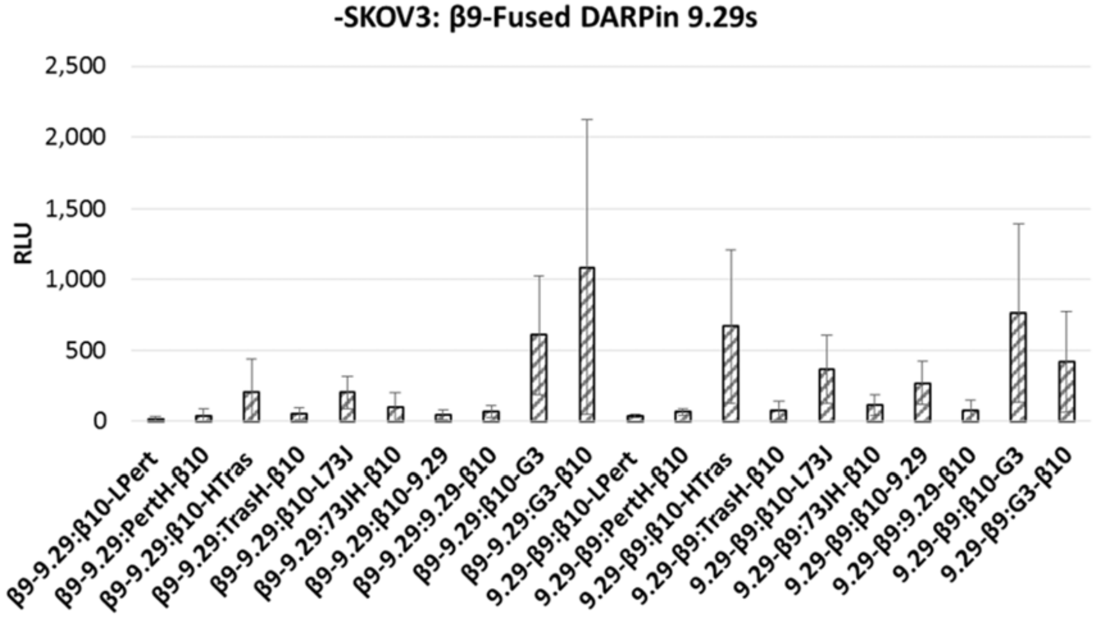
-SKOV3: β9-Fused DARPin 9.29s
RLU
2,500
2,000
1,500
1,000
500
0
β9-9.29:β10-LPert
β9-9.29:PertH-β10
β9-9.29:β10-HTras
β9-9.29:TrasH-β10
β9-9.29:β10-L73J
β9-9.29:73JH-β10
β9-9.29:β10-9.29
β9-9.29:9.29-β10
β9-9.29:β10-G3
β9-9.29:G3-β10
9.29-β9:β10-LPert
9.29-β9:PertH-β10
9.29-β9:β10-HTras
9.29-β9:TrasH-β10
9.29-β9:β10-L73J
9.29-β9:73JH-β10
9.29-β9:β10-9.29
9.29-β9:9.29-β10
9.29-β9:β10-G3
9.29-β9:G3-β10

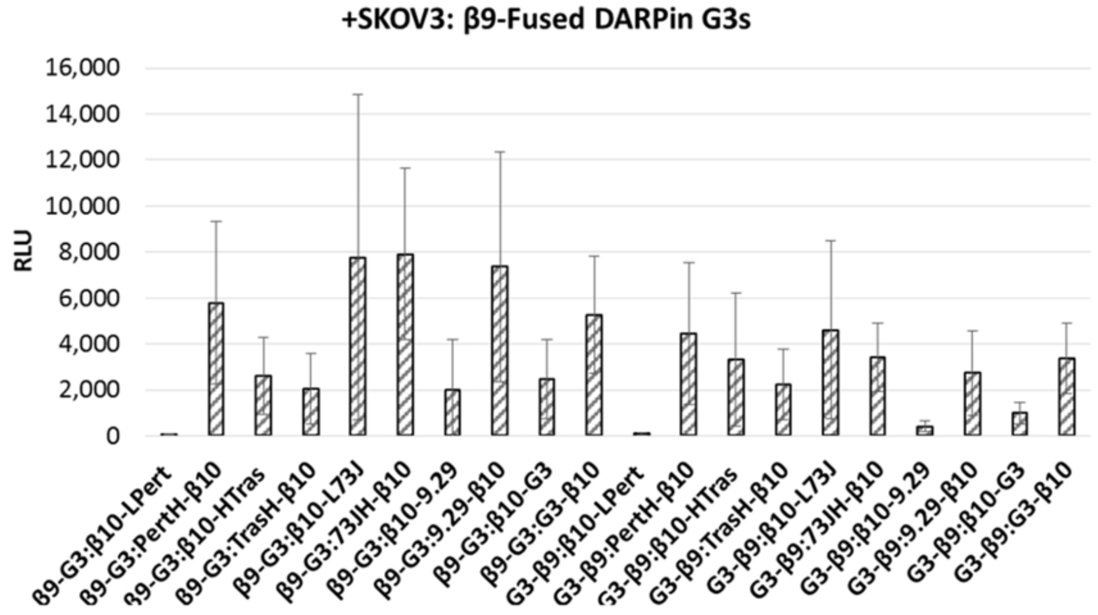

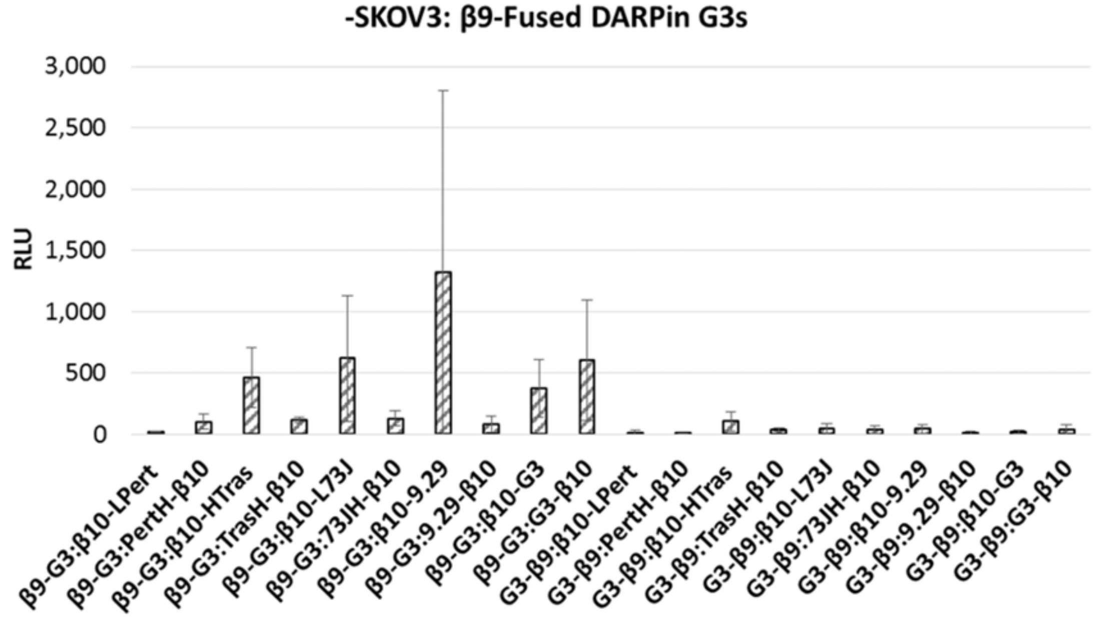

Figure S5. TEC signal produced from each of 100 pairs on SKOV3 cells, or empty wells. The average values from three experiments performed on three separate days are represented with the standard deviation indicated by the error bars. The majority of the pairs did not result in luminescence above the instrumental noise (≈100 RLU) in the absence of the HER2-expressing SKOV3 cells.

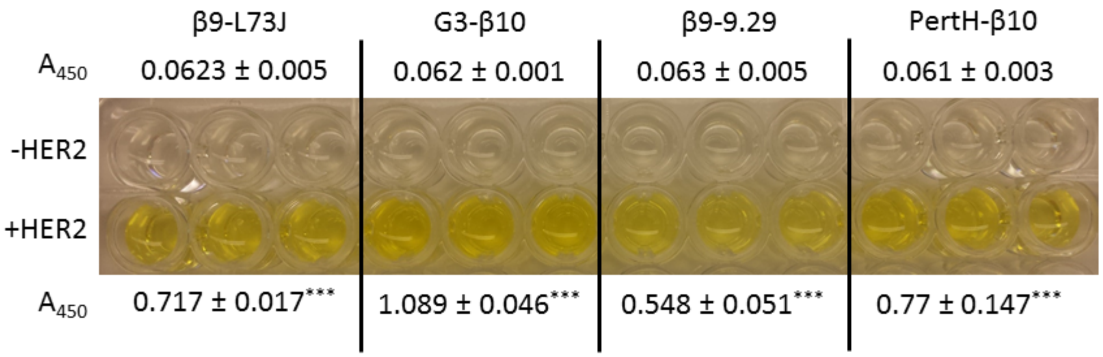

Figure S6. Verification the purified fusions retain HER2 binding. After adsorbing PBS (-HER2) or recombinant HER2 (+HER2) to the wells of an ELISA plate, an indirect ELISA was performed using the purified fusions as the primary antibody. The fusions were detected by addition of a mouse anti-His antibody followed by an anti-mouse HRP conjugated antibody. Each binder was tested with and without HER2 in triplicate as shown in the image, and the average absorbance (± S.D.) is indicated above (-HER2) or below (+HER2) the image.

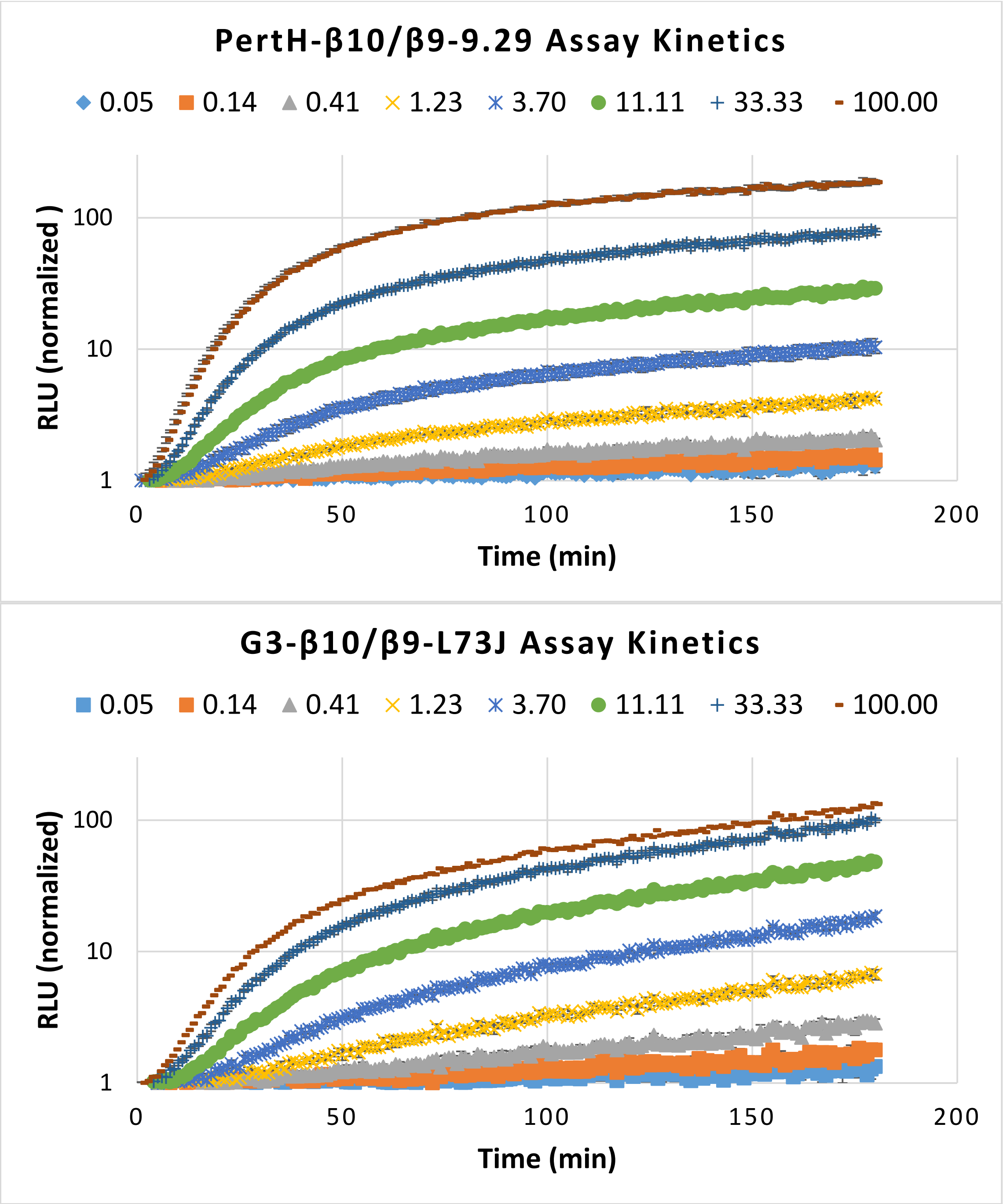

Figure S7. Signal kinetics from G3-β10/β9-L73J (A) or PertH-β10/β9-9.29 (B) with various concentrations of HER2. Assay details are as described for figure 5A-B.

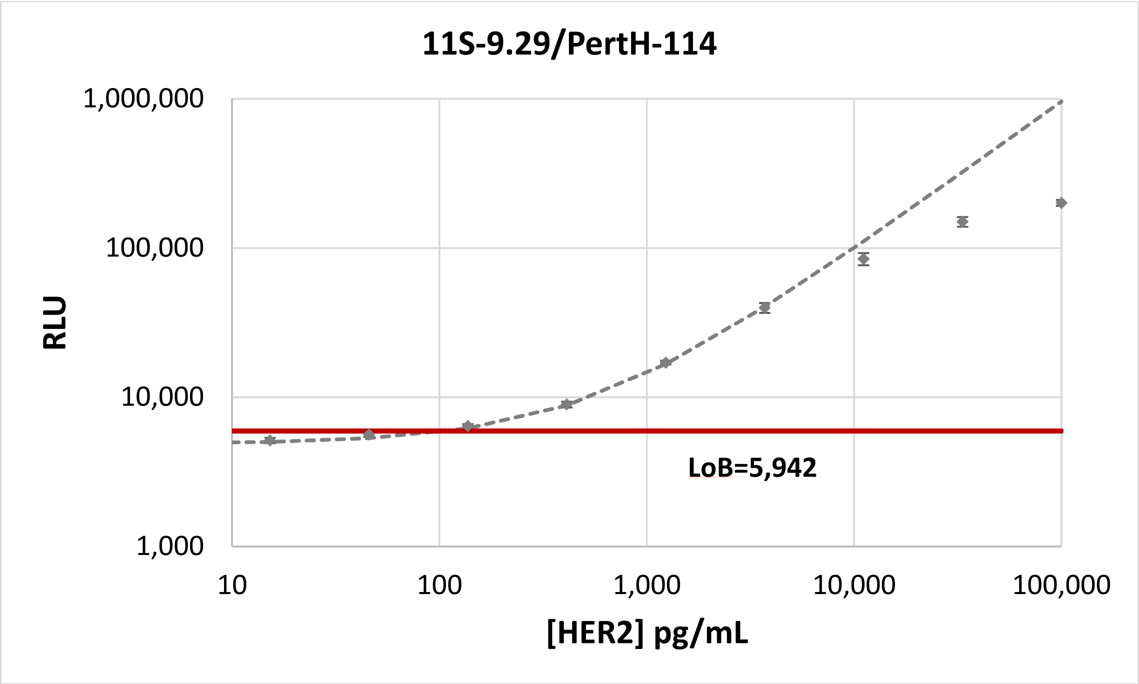

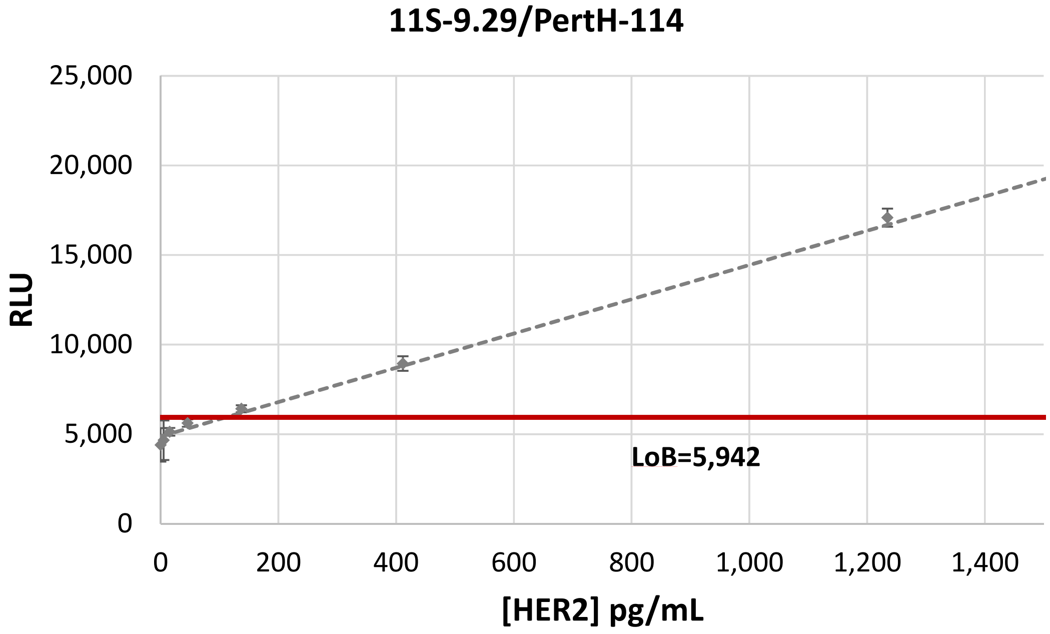

Figure S8. TEC using NanoBiT as complementation system. 11S-9.29 and PertH-114 lysates were used at 1:10^4 dilutions with 10 μM Nano-Glo substrate. Measurements shown are 2 hrs after addition of HER2.

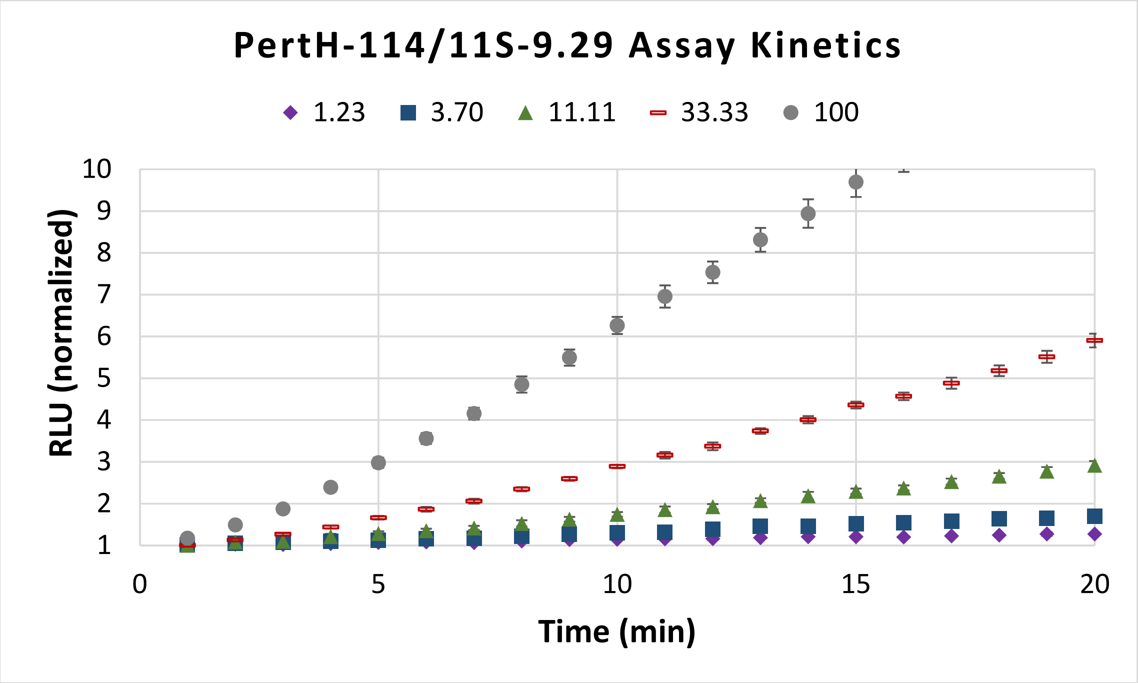

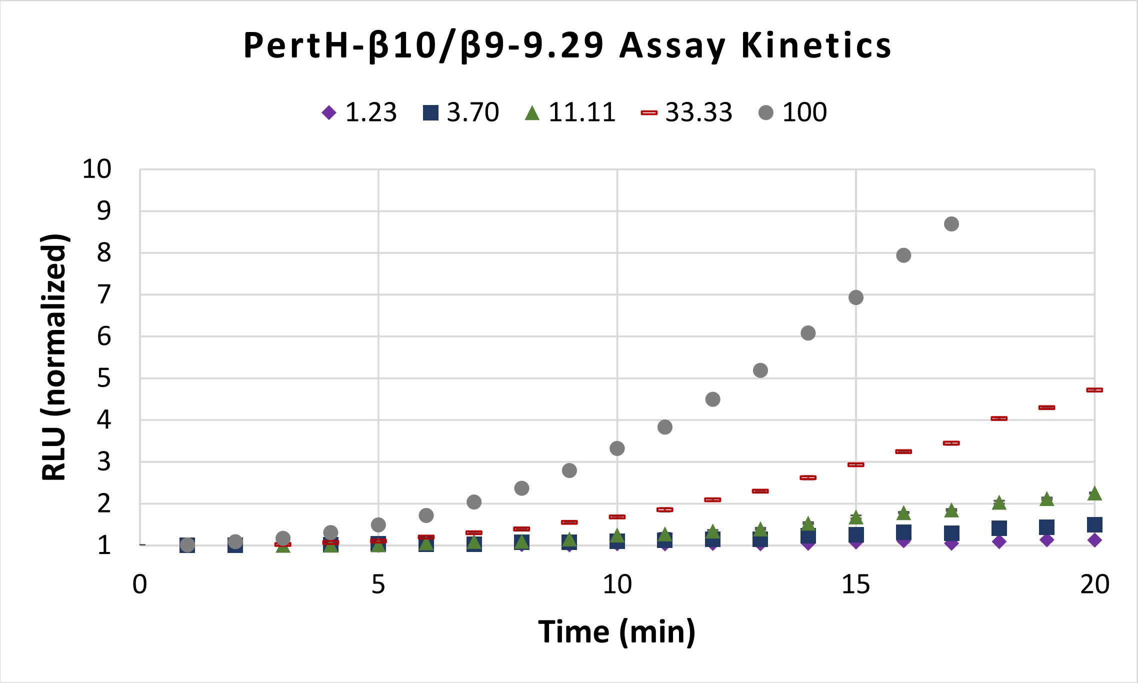

Figure S9. Comparison of assay kinetics for binary (A) and ternary (B) systems. 11S-9.29 and PertH-114 lysates were used at 1:10^4 dilutions with 10 µM Nano-Glo substrate. Measurements shown are 2 hrs after addition of indicated concentrations of HER2, ranging from 1.23 to 100 ng/mL.

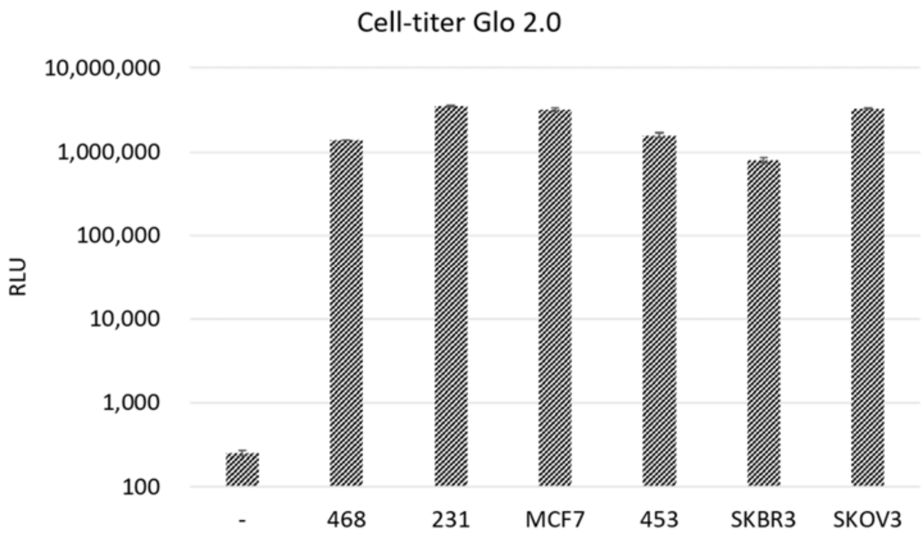

Figure S10. Quantification of viable cells at the time of performing the TEC assay (1-day after seeding the cells). Data shown is the average values from three separate wells with the standard deviation indicated by the error bars.

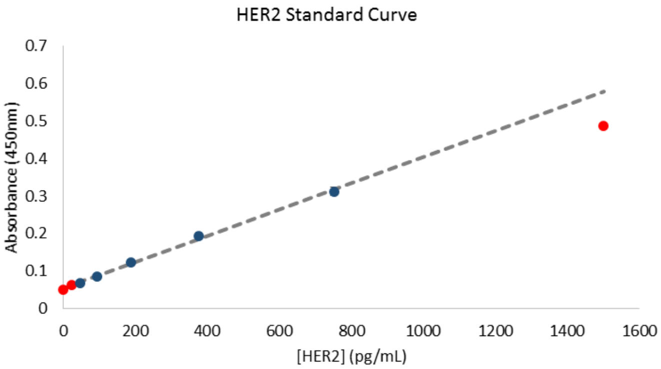

Dilution Factor	A (450nm)	S.D.	pg/mL	pg/mL x Dil. Fact.
160	0.0732	0.0030	55	8,770
80	0.0969	0.0032	122	9,788
40	0.1429	0.0019	254	10,145
20	0.2346	0.0130	515	10,306
			[HER2] pg/mL	9,752

Figure S11. Quantification of HER2 in human serum through sandwich ELISA. The dashed line, y = 0.00035x + 0.054, was found by fitting the data indicated with blue circles, and linear range determined to be within the absorbance values of 0.0679 – 0.3118 resulting from 50 – 800 pg/mL HER2 (data points in red are not within this range). Human serum was serially diluted, and dilutions yielding absorbance values within the linear range (table on right) were used in calculating the HER2 concentration. The concentration of HER2 in the serum was determined by averaging the calculated concentration from each of these dilutions, and found to be 9.752 ng/mL.

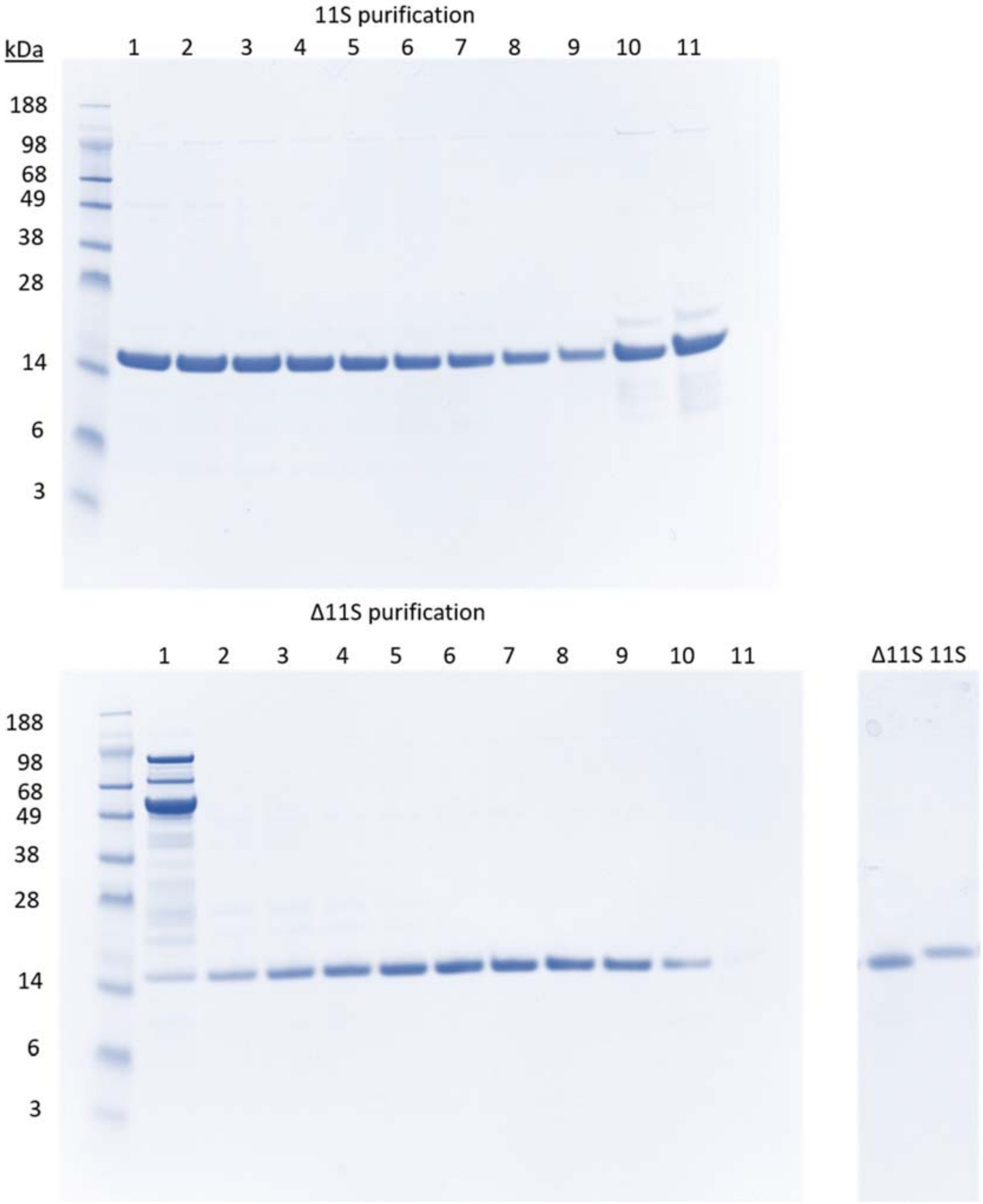

Figure S12. SDS-PAGE of 11S and Δ11S purifications. 11S and Δ11S were expressed in E. coli and purified via IMAC using imidazole to elute the His-tagged protein. 11S Purification: lanes 1-9 elution fractions from ≈35 mM - 55 mM imidazole with ≈2 mM increase per fraction, lanes 10-11 elution fractions with 200 mM imidazole. Δ11S Purification: lanes 1-10 elution fractions from ≈20 mM – 60 mM imidazole with ≈3 mM increase per fraction, lane 11 elution with 200 mM imidazole.

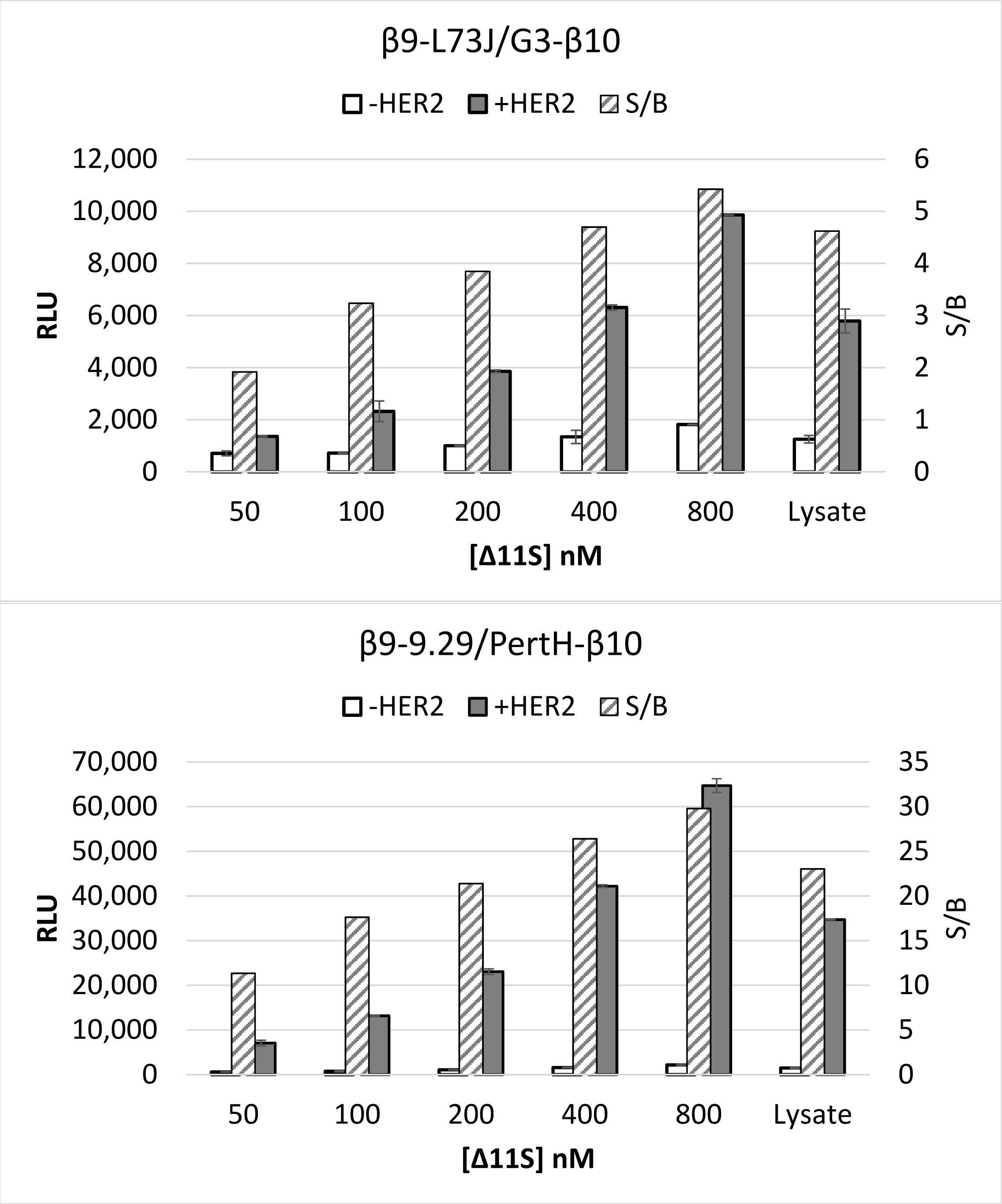

Figure S13. TEC results from various concentrations of purified Δ11S or a lysate of Δ11S. Complete detection solutions were used: 2 nM β9-L73J and G3-β10 or 1.5 nM β9-9.29 and PertH-β10, 10 μM Nano-Glo substrate, and indicated concentration of Δ11S (Δ11S lysate diluted 1:20). 25 μL of 2X detection solution was mixed with 25 μL of 1 ng/mL HER2 and luminescence measured at 2 hrs.

Table T4. List of primers used for cloning the genetic fusion constructs.

Clone	Primer #	Fw/ Rev	Name	Sequence
Δ11S	1	Fw	T7 Fw (long)	5'-CGATCCCGCGAAATTAATACGACTCACTATAGGG-3'

	2	Rev	11S 146 Rev (+Xba)	5'-GCAGGTCGACTCTAGATTAATCCGGTGTAATCAGACGTTCATCGATAATTTTGTTACC-3'
β10Δ11S	3	Fw	11S Fw (+linker)	5'-GTGGTGGTAGCGGTGGTGGTGGTAGCGGTATGGTTTTTACCCTGGAAGATTTTGTGGGTG-3'
	4	Fw	10 G/S Linker Fw (+β10)	5'-ATCATGTGAGCGGTTGGCGTCTGTTCAAAAAAATCAGCGGTGGTGGTAGCGGTGGTGGTG-3'
	5	Fw	β10 Fw (+H6)	5'-TTAGTAAGGAGCGATCGCCATGCATCATCATCACCATCATGTGAGCGGTTGGCGTCTG-3'
	6	Rev	11S 146 Rev (+Xba)	5'-GCAGGTCGACTCTAGATTAATCCGGTGTAATCAGACGTTCATCGATAATTTTGTTACC-3'
VL	7	Fw	Variable LC Fw	5'-ATGGCAAGCGATATTCAGATGACCCAGAGC-3'
	8	Rev	Variable LC Rev	5'-GGCGGAAAGATAAAAACGCTCGGTGCTGCAACGGTACGTTTAATTTCAACTTTGGTGCCC-3'
CL	9	Fw	Const LC Fw (StoE)	5'-GCACCGAGCGTTTTTATCTTTCCGCCTAGCGATGAGCAGCTGAAAAGCGGTACAGCCAGC-3'
	10	Rev	Int Rev	5'-CCATAAAATCACCTCAACCTCTAGTTACCCTTTTTACGTG-3'
VH	11	Fw	Variable HC Fw	5'-GAAATTAGCGAAGTTCAGCTGGTTGAAAGCG-3'
	12	Rev	Variable HC Rev	5'-GCTGCTAACGGTAACCAGGGTGC-3'
β9-VH-CH	13	Fw	β9 Fw (Int)	5'-GGGTAACTAGAGGTTGAGGTGATTTTATGGGTAGTATGCTGTTTCGTGTTACCATTAAC-3'
	14	Rev	Variable HC Rev	5'-GCTGCTAACGGTAACCAGGGTGC-3'
β10-VH-CH	15	Fw	β10 Fw (+Int)	5'-GGGTAACTAGAGGTTGAGGTGATTTTATGGTGAGCGGTTGGCGTCTG-3'
	16	Rev	Variable HC Rev	5'-GCTGCTAACGGTAACCAGGGTGC-3'
CH1	17	Fw	Const1 HC Fw	5'-GCACCCTGGTTACCGTTAGCAGC-3'
	18	Rev	Heavy Rev	5'-GCAGGTCGACTCTAGATTAGGTATGGGTTTTATCGCAGCTTTTCGGTTC-3'
VL-CL	19	Fw	Variable LC Fw	5'-ATGGCAAGCGATATTCAGATGACCCAGAGC-3'
	20	Fw	Variable LC Fw (+Sgf)	5'-TTAGTAAGGAGCGATCGCCATGGCAAGCGATATTCAGATGACCCAGAG-3'
	21	Rev	Int Rev	5'-CCATAAAATCACCTCAACCTCTAGTTACCCTTTTTACGTG-3'
β9-VL-CL	22	Fw	VL Fw (+H8)	5'-CCATCACCATCATGGTGGTGGCAGTGGTATGGCAAGCGATATTCAGATGACCCAGAG-3'
	23	Fw	H8 Fw (+β9)	5'-TTAGTAAGGAGCGATCGCCATGGGTAGTATGCTGTTTCGTGTTACCATTAACAGCGGTGGTGGTAGCGGTCATCATCACCACCATCACCATCATGGTGGTGGCAG-3'
	24	Rev	Int Rev (+HCV)	5'-CTGAACTTCGCTAATTTCCATAAAATCACCTCAACCTCTAGTTACCCTTTTTACGTG-3'

β10-VL-CL	25	Fw	VL Fw (+H8)	5'-CCATCACCATCATGGTGGTGGCAGTGGTATGGCAAGCGATATTCAGATGACCCAGAG-3'
	26	Fw	H8 Fw (+β10)	5'-TTAGTAAGGAGCGATCGCCATGGTGAGCGGTTGGCGTCTGTTCAAAAAAATCAGCGGTGGTGGTAGCGGTCATCATCACCACCATCACCATCATGGTGGTGGCAG-3'
	27	Rev	Int Rev (+HCV)	5'-CTGAACTTCGCTAATTTCCATAAAATCACCTCAACCTCTAGTTACCCTTTTTACGTG-3'
VH-CH1	28	Fw	Variable HC Fw	5'-GAAATTAGCGAAGTTCAGCTGGTTGAAAGCG-3'
	29	Fw	Variable HC Fw (+int)	5'-GAGGTTGAGGTGATTTTATGGAAATTAGCGAAGTTCAGCTGGTTGAAAGCG-3'
	30	Rev	Heavy Rev	5'-GCAGGTCGACTCTAGATTAGGTATGGGTTTTATCGCAGCTTTTCGGTTC-3'
β9-VH-CH	31	Fw	β9 Fw (Int)	5'-GGGTAACTAGAGGTTGAGGTGATTTTATGGGTAGTATGCTGTTTCGTGTTACCATTAAC-3'
	32	Rev	Heavy Rev	5'-GCAGGTCGACTCTAGATTAGGTATGGGTTTTATCGCAGCTTTTCGGTTC-3'

Table T4 (cont). List of primers used for cloning the genetic fusion constructs.

Clone	Primer #	Fw/ Rev	Name	Sequence
β10-VH-CH	33	Fw	β10 Fw (+Int)	5'-GGGTAACTAGAGGTTGAGGTGATTTTATGGTGAGCGGTTGGCGTCTG-3'
	34	Rev	Heavy Rev	5'-GCAGGTCGACTCTAGATTAGGTATGGGTTTTATCGCAGCTTTTCGGTTC-3'
VH-CH1-β9	35	Fw	Variable HC Fw (+int)	5'-GAGGTTGAGGTGATTTTATGGAAATTAGCGAAGTTCAGCTGGTTGAAAGCG-3'
	36	Rev	CH rev (link)	5'-GGTGATGATGACCGCTACCACCACCGGTATGGGTTTTATCGCAGCTTTTCGGTTC-3'
	37	Rev	H8 rev (+β9)	5'-GCAGGTCGACTCTAGATTAGCTGTTAATGGTAACACGAAACAGCATACTACCACCACTGCCACCACCATGATGGTGATGGTGGTGATGATGACCGCTACCACCACC-3'
VH-CH1-β9	38	Fw	Variable HC Fw (+int)	5'-GAGGTTGAGGTGATTTTATGGAAATTAGCGAAGTTCAGCTGGTTGAAAGCG-3'
	39	Rev	CH rev (link)	5'-GGTGATGATGACCGCTACCACCACCGGTATGGGTTTTATCGCAGCTTTTCGGTTC-3'
	40	Rev	H8 rev (+β10)	5'-GCAGGTCGACTCTAGATTAGCTGATTTTTTTGAACAGACGCCAACCGCTCACACCACTGCCACCACCATGATGGTGATGGTGGTGATGATGACCGCTACCACCACC-3'
β9-G3	41	Fw	G3 Fw (+5link)	5'-ACCATCACCATCATGGTGGTGGCAGTGGTGATCTGGGTAAAAAACTGCTGGAAGCAGC-3'

	42	Fw	H8 Fw (+β9)	5'-TTAGTAAGGAGCGATCGCCATGGGTAGTATGCTGTTTCGTGTTACCATTAACAGCGGTGGTGGTAGCGGTCATCATCACCACCATCACCATCATGGTGGTGGCAG-3'
	43	Rev	pF1K Rev	5'-AGCTTGCATGCCTGCAGGTCGAC-3'
β10-G3	44	Fw	G3 Fw (+5link)	5'-ACCATCACCATCATGGTGGTGGCAGTGGTGATCTGGGTAAAAAACTGCTGGAAGCAGC-3'
	45	Fw	H8 Fw (+β10)	5'-TTAGTAAGGAGCGATCGCCATGGTGAGCGGTTGGCGTCTGTTCAAAAAAATCAGCGGTGGTGGTAGCGGTCATCATCACCACCATCACCATCATGGTGGTGGCAG-3'
	46	Rev	pF1K Rev	5'-AGCTTGCATGCCTGCAGGTCGAC-3'
G3-β9	47	Fw	T7 Fw (long)	5'-CGATCCCGCGAAATTAATACGACTCACTATAGGG-3'
	48	Rev	G3 Rev (+15link)	5'-GCCACCTGAGCCACCACCACCGCTACCACCACCGTTCAGTTTCTGCAGGATTTCTGCCAG-3'
	49	Rev	15link Rev (+β9)	5'-GCAGGTCGACTCTAGATTAGCTGTTAATGGTAACACGAAACAGCATACTACCACCAGAACCACCGCCACCTGAGCCACCACCAC-3'
G3-β10	50	Fw	T7 Fw (long)	5'-CGATCCCGCGAAATTAATACGACTCACTATAGGG-3'
	51	Rev	G3 Rev (+15link)	5'-GCCACCTGAGCCACCACCACCGCTACCACCACCGTTCAGTTTCTGCAGGATTTCTGCCAG-3'
	52	Rev	15link Rev (+β10)	5'-GCAGGTCGACTCTAGATTAGCTGATTTTTTTGAACAGACGCCAACCGCTCACACCAGAACCACCGCCACCTGAGCCACCACCAC-3'
β9-9.29	53	Fw	9.29 Fw (+link)	5'-CCATCACCATCATGGTGGTGGCAGTGGTGGTAGCGATCTGGGTAAAAAACTGCTGG-3'
	54	Fw	H8 Fw (+β9)	5'-TTAGTAAGGAGCGATCGCCATGGGTAGTATGCTGTTTCGTGTTACCATTAACAGCGGTGGTGGTAGCGGTCATCATCACCACCATCACCATCATGGTGGTGGCAG-3'
	55	Rev	9.29 Rev (+Xba)	5'-GCAGGTCGACTCTAGATTATGCTGCTTTCTGCAGCACCTCG-3'
β10-9.29	56	Fw	9.29 Fw (+link)	5'-CCATCACCATCATGGTGGTGGCAGTGGTGGTAGCGATCTGGGTAAAAAACTGCTGG-3'
	57	Fw	H8 Fw (+β10)	5'-TTAGTAAGGAGCGATCGCCATGGTGAGCGGTTGGCGTCTGTTCAAAAAAATCAGCGGTGGTGGTAGCGGTCATCATCACCACCATCACCATCATGGTGGTGGCAG-3'
	58	Rev	9.29 Rev (+Xba)	5'-GCAGGTCGACTCTAGATTATGCTGCTTTCTGCAGCACCTCG-3'
9.29-β9	59	Fw	9.29 Fw (+Sgf)	5'-TTAGTAAGGAGCGATCGCCATGGGTAGCGATCTGGGTAAAAAACTGCTGG-3'
	60	Rev	9.29 Rev (link)	5'-GGTGATGATGACCGCTACCACCACCTGCTGCTTTCTGCAGCACCTCG-3'
	61	Rev	H8 rev (+β9)	5'-GCAGGTCGACTCTAGATTAGCTGTTAATGGTAACACGAAACAGCATACTACCACCACTGCCACCACCATGATGGTGATGGTGGTGATGATGACCGCTACCACCACC-3'

9.29-β10	62	Fw	9.29 Fw (+Sgf)	5'-TTAGTAAGGAGCGATCGCCATGGGTAGCGATCTGGGTAAAAAACTGCTGG-3'
	63	Rev	9.29 Rev (link)	5'-GGTGATGATGACCGCTACCACCACCTGCTGCTTTCTGCAGCACCTCG-3'
	64	Rev	H8 rev (+β10)	5'-GCAGGTCGACTCTAGATTAGCTGATTTTTTTGAACAGACGCCAACCGCTCACACCACTGCCACCACCATGATGGTGATGGTGGTGATGATGACCGCTACCACCACC-3'
